# Can universal cervical length screening with vaginal progesterone for a short cervix reduce preterm birth? A systematic review and meta‐analyses

**DOI:** 10.1111/aogs.70253

**Published:** 2026-05-20

**Authors:** Mira Zethelius, Lina Bergman, Ann‐Catrin Ekelund, Cecilie Hongslo Vala, Bo Jacobsson, Pihla Kuusela, Ann Liljegren, Björn Lindkvist, Max Petzold, Petteri Sjögren, Ulla‐Britt Wennerholm, Tove Wikström

**Affiliations:** ^1^ Department of Gynaecology and Obstetrics South Älvsborg Hospital Borås Sweden; ^2^ Department of Obstetrics and Gynaecology Sahlgrenska University Hospital Gothenburg Sweden; ^3^ Department of Obstetrics and Gynaecology, Institute of Clinical Sciences, Sahlgrenska Academy University of Gothenburg Gothenburg Sweden; ^4^ Medical Library Skaraborg Hospital Skövde Sweden; ^5^ Center for Health Technology Assessment Sahlgrenska University Hospital Gothenburg Sweden; ^6^ Department of Obstetrics and Gynaecology Sahlgrenska University Hospital/Östra Hospital Gothenburg Sweden; ^7^ Medical Library Sahlgrenska University Hospital Gothenburg Sweden; ^8^ School of Public Health and Community Medicine, Institute of Medicine University of Gothenburg Gothenburg Sweden

**Keywords:** cervical length, preterm birth, progesterone, short cervix, transvaginal ultrasound, universal screening

## Abstract

**Introduction:**

The global rate of preterm birth (PTB) is not declining, resulting in approximately one million deaths annually among children under five due to complications related to PTB. Universal cervical length screening followed by progesterone treatment in women with a short cervix has been proposed as a preventive strategy. We systematically assessed the accuracy of this approach in reducing the incidence of PTB and improving neonatal outcomes.

**Material and Methods:**

We conducted a systematic literature search and meta‐analysis registered in PROSPERO (CRD42024605203). Medline, Embase, and the Cochrane Library were searched in October 2024 and updated in September 2025. Eligible studies included randomized controlled trials (RCTs), cohort studies, and systematic reviews comparing universal cervical length screening with vaginal ultrasound in mid‐trimester followed by progesterone treatment versus no screening in asymptomatic women with a singleton pregnancy. Outcomes of interest were any PTB, spontaneous PTB (sPTB), and neonatal outcomes. The certainty of evidence was assessed separately for RCTs and cohort studies using the GRADE approach.

**Results:**

Two RCTs (1634 participants) and four cohort studies (425 735 participants) were included. Results from RCTs showed no statistically significant reduction in any PTB, sPTB, peri/neonatal mortality, or morbidity after screening versus no screening. In contrast, meta‐analyses of cohort studies demonstrated a significant reduction for any PTB < 37 weeks; adjusted odds ratio 0.87, 95% confidence interval (CI) 0.78–0.98, and sPTB < 37 weeks; relative risk 0.82, 95% CI 0.70–0.96. Meta‐analyses of cohort studies also demonstrated a significant reduction in sPTB < 34 and <32 weeks. The cohort studies presented no data on neonatal outcomes. The certainty of evidence from RCTs was rated very low due to limited precision and lack of significant results. Although meta‐analyses of cohort studies showed significant associations, the certainty of evidence was also very low because of indirectness and a high risk of bias inherent in observational study designs.

**Conclusions:**

It is uncertain, based on very low certainty of evidence, whether universal cervical length screening followed by progesterone treatment for women with a short cervix reduces PTB or affects neonatal outcomes, compared with no screening. More adequately powered trials are needed before a recommendation can be made.

## Graphical abstract

Preterm birth is associated with substantial neonatal morbidity and mortality. We evaluated whether universal cervical length screening with ultrasound, followed by progesterone therapy for women with a short cervix, has the potential to reduce the incidence of preterm birth. We found that current evidence is insufficient to recommend universal screening.

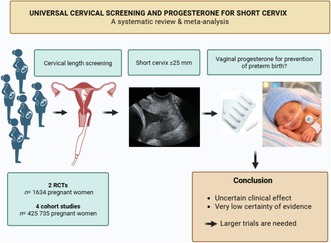

**Abbreviations**
aOR: adjusted odds ratio
CI: confidence interval
CL: cervical length
FIGO: International Federation of Gynecology and Obstetrics
GRADE: Grading of Recommendations, Assessment Development and Evaluations
HTA: Health Technology Assessment
ISUOG: The International Society of Ultrasound in Obstetrics & Gynecology
n: number
n.s.: non‐significant
OR: odds ratio
PICO: population intervention comparison outcome
PRISMA: Preferred Reporting Items for Systematic Reviews and Meta‐Analyses
PROSPERO: The International Prospective Register of Systematic Reviews
PTB: preterm birth
RCT: randomized controlled trial
RoB 2: Revised Cochrane risk‐of‐bias tool for RCTs
ROBINS‐I: Risk Of Bias In Non‐randomized Studies
RR: risk ratio
SBU: Swedish Agency for Health Technology Assessment and Assessment of Social Services
SOF: summary of findings
sPTB: spontaneous preterm birth
SR: systematic review
w: weeks

**Key message**

Babies born preterm face a high risk of complications. We evaluated whether universal cervical screening with ultrasound and progesterone for women with a short cervix might prevent preterm birth. We found that current evidence is insufficient to recommend universal screening.

## 1 INTRODUCTION

Preterm birth (PTB), defined as birth before 37 completed weeks, remains a major global health concern. Despite advances in maternal care, the global rate of PTB has not declined.^1^

In 2020, 13.4 million children were born preterm in the world.^2^ This corresponds to 10% of all newborns, and more than 40% of these children will suffer complications during the neonatal period,^3^ contributing to approximately one million deaths annually among children under five.^4^ Furthermore, survivors of PTB face a higher risk of both short‐and long‐term complications.^5^, ^6^ In addition to the apparent suffering, PTB represents a high cost to society, from intensive, short‐term neonatal care to long‐term ongoing support.^7^

PTB is commonly subdivided into spontaneous PTB (sPTB) and indicated PTB (induced birth because of fetal or maternal complications). sPTB is a syndrome that accounts for approximately two‐thirds of all PTB and has been associated with a wide range of pathological processes.^8^ There are several reproductive, maternal, and gestational risk factors associated with sPTB, where one or repeated sPTBs show the strongest association. However, 50% of women giving birth preterm have no identifiable risk factor.^8^ Screening methods, such as biomarkers or cervical length screening, are also important to identify women without known risk factors of PTB.^9^

Numerous studies have shown that a short cervix measured by transvaginal ultrasound is a strong risk factor for PTB.^10^, ^11^, ^12^ However, the low prevalence in a low‐risk population (0.5%–7.9%)^13^, ^14^, ^15^ and the moderate predictive accuracy of transvaginal cervical ultrasound question the usefulness of universal screening. Furthermore, the optimal cut‐off value for defining a “short cervix” remains controversial.^11^ The implementation of universal screening for all women with singleton pregnancies thus remains a subject of ongoing debate.^10^, ^16^, ^17^, ^18^, ^19^

Progesterone is the most promising available preventive treatment for women at increased risk of PTB.^20^ Meta‐analyses show that vaginal progesterone treatment reduces the rate of any PTB by 22%–38%, with the most robust results for PTB before 33 weeks.^20^, ^21^, ^22^ In addition, women at low risk (without previous sPTB) seem to benefit from progesterone treatment. Furthermore, cost‐effectiveness studies consistently indicate that cervical length screening of asymptomatic women with a singleton pregnancy, followed by progesterone treatment for those identified as at risk, is a cost‐effective strategy.^7^, ^23^, ^24^, ^25^

This systematic review (SR) aimed to evaluate the effectiveness of universal cervical length screening with transvaginal ultrasound in asymptomatic women with singleton pregnancies, followed by progesterone treatment for those with a short cervix, in reducing the incidence of PTB and improving perinatal outcomes.

## 2 MATERIAL AND METHODS

This study follows the Preferred Reporting Items for Systematic Reviews and Meta‐Analyses (PRISMA) guidelines^26^ (Table S1). The study was prospectively registered in The International Prospective Register of Systematic Reviews (PROSPERO; CRD42024605203, available at https://www.crd.york.ac.uk/PROSPERO/view/CRD42024605203) on November 7, 2024, prior to data extraction.

### 2.1 Search strategy

Systematic literature searches were conducted in Medline, Embase, and the Cochrane Library on October 24, 2024, with an updated search on September 24, 2025, by two authors (AL, ACE). The search strategy combines controlled vocabulary and keyword terms related to the PICO (see the Section 2.2). In addition, websites of Scandinavian national and regional health technology assessment (HTA) organizations were visited on October 14, 2024. Reference lists of relevant reports were scrutinized for additional references. A search for ongoing trials in Clinicaltrials.gov (Dec 13, 2024) identified 54 trials, and in the WHO International Clinical Trials Registry Platform (Dec 13, 2024) identified 18 trials, none of which met our inclusion criteria. A detailed search strategy is presented in Table S2.

### 2.2 Study selection criteria

Articles were included according to a predefined PICO (population, intervention, comparison, outcome); see Table S3. The population (P) was defined as asymptomatic women (no signs of preterm labor) with singleton pregnancies, intervention (I) as transvaginal ultrasound cervical length screening in the first or second trimester, and treatment with vaginal or oral progesterone, with or without additional treatment, if a short cervix was demonstrated (as defined by the authors). The comparison group (C) was defined as women with a singleton pregnancy who were not screened in the first or second trimester.

Main outcomes (O) were any PTB or sPTB with any cut‐off below 37 + 0 weeks, perinatal mortality (intrauterine fetal death and neonatal mortality <7 or <28 days), neonatal mortality (<7 or <28 days), serious neonatal morbidity (such as bronchopulmonary dysplasia, severe intraventricular hemorrhage, necrotizing enterocolitis, confirmed sepsis, retinopathy of prematurity), individually or as a composite outcome with or without peri/neonatal mortality.

Randomized controlled trials (RCTs) of any size and cohort studies with at least 1000 screened women from 1980 and SRs from 2020 written in Danish, English, Norwegian, or Swedish were eligible for inclusion. Articles were excluded if they did not fulfill the above‐mentioned inclusion and eligibility criteria.

### 2.3 Data extraction

The authors were selected specifically for this review as experts in this field (LB, BJ, PK, UBW, TW, MP, MZ), as experts of HTA (BL, CVH, PS), and as medical librarians, experts in systematic literature searches (AL, ACE). AL and ACE independently assessed the retrieved abstracts and made the first selection of full‐text articles for inclusion. The selected articles were sent to all authors. In a consensus meeting, experts in the field and experts in HTA decided which articles to include. Any disagreements were resolved in consensus. Data were extracted by at least two authors independently and cross‐checked by two different authors. Extracted data included title, authors, year and country, study design, study period, number of participants, timing of transvaginal ultrasound, cervical length cut‐off, inclusion and exclusion criteria, interventions for prevention of PTB, concomitant intervention, and outcome variables. The reference list of included articles is presented in Table S4, and excluded articles with reasons for exclusion in Table S5.

### 2.4 Critical appraisal, data synthesis, and certainty of evidence

All experts in the field and of HTA critically appraised the included studies independently for directness, risk of bias, and precision using checklists, the Revised Cochrane risk‐of‐bias tool (RoB 2) for RCTs, and Risk Of Bias In Non‐randomized Studies (ROBINS‐I) for cohort studies, modified by the Swedish Agency for Health Technology Assessment and Assessment of Social Services (SBU).

Data were summarized for each outcome in all the included studies that met the criteria in the predefined PICO. When possible, data were pooled in meta‐analysis (RevMan 5.4 and Stata 19) using the random effect and fixed effect models and RR as the point estimate with 95% CI. When available, the adjusted odds ratio (aOR) was used. RCTs and cohort studies were handled separately in the meta‐analyses.^27^ In case of low event rates (<1%) in one arm, the Peto odds ratio (OR) was used.^28^ Heterogeneity between studies was explored by calculating the I^2^ value. In meta‐analysis with fewer than five studies, the I^2^ value was assessed qualitatively due to large uncertainty. The different aspects were categorized as no/minor, some, or major problems. Disagreements were resolved in consensus.

The certainty of evidence for each outcome was assessed using the GRADE approach separately for RCTs and cohort studies^27^ and was critically appraised by all authors. Disagreements were solved in consensus. The GRADE worksheet includes several domains: summary risk of bias (random sequence generation, allocation concealment, blinding of participants and personnel, blinding of outcome assessment, incomplete outcome data, selective reporting, other bias), directness, precision, study limitations, consistency/heterogeneity, publication bias (not used if fewer than five studies), and magnitude of effect. The GRADE rating results in an assessment of the certainty of evidence in four grades: high, moderate, low, and very low (see footnote Table 3, Summary of findings).

### 2.5 Subgroup analyses

Planned subgroup and sensitivity analyses were performed using different cut‐offs for cervical length, women with or without previous PTB, first‐trimester or second‐trimester screening, and exclusion of studies where the treatment included more than only progesterone.

## 3 RESULTS

### 3.1 Study selection

**FIGURE 1:**
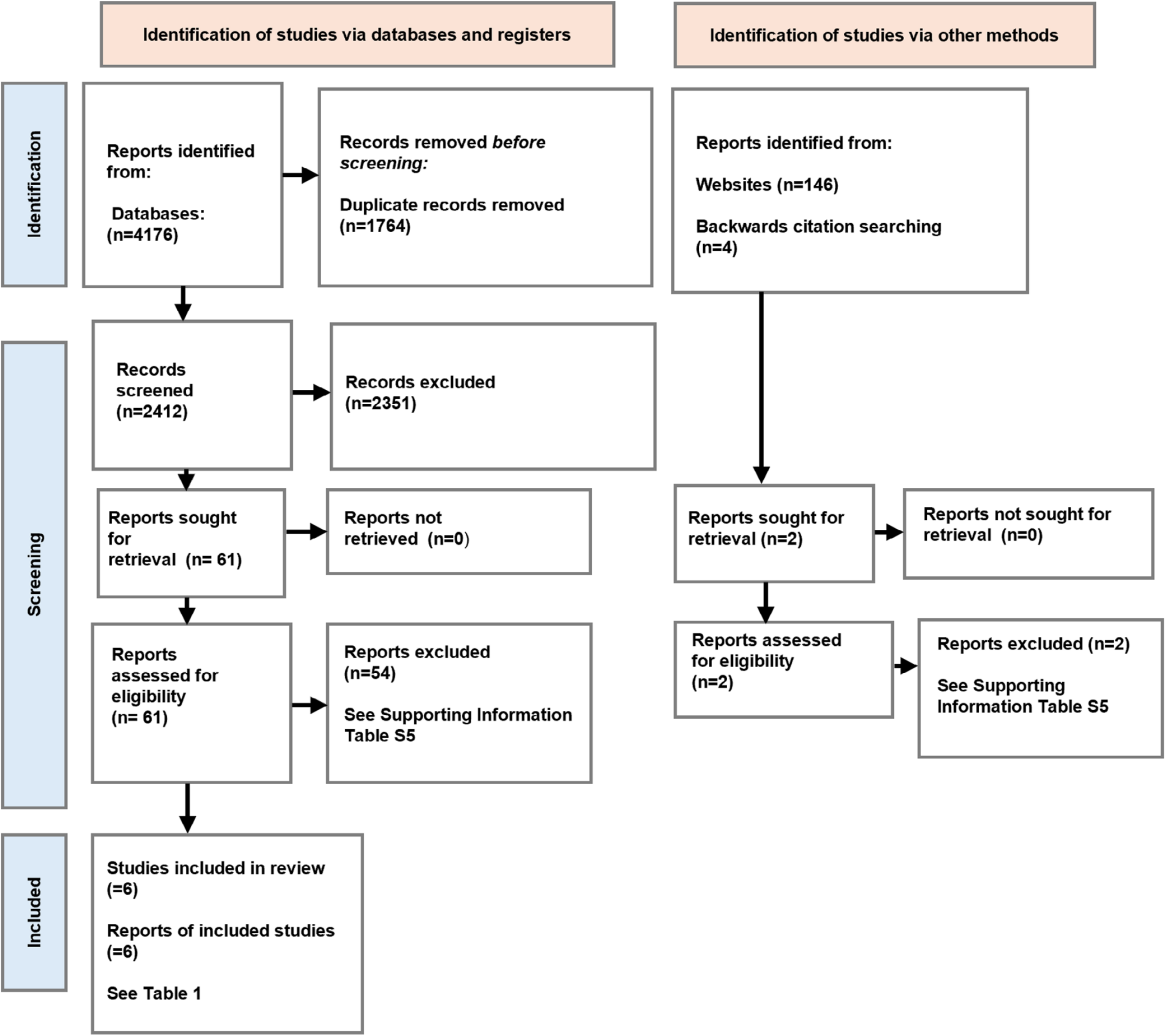
PRISMA flow diagram of the selection process for systematic reviews.

The literature search performed in October 2024 and September 2025 identified 2412 records after removal of duplicates (Figure 1). DedupEndNote was used for deduplication. All abstracts were screened using the Rayyan tool,^29^ a SR software. After reading the abstracts, 2351 records were excluded, and 61 articles were sought for retrieval. A total of 31 of these publications were excluded by two authors (AL, ACE) after reading them in full text. The remaining 32 publications were sent to all participants of the project group. Of these, 26 publications were excluded after full‐text reading and discussion in the project's consensus meetings. Six publications were finally included in the assessment: two RCTs^30^, ^31^ and four retrospective cohort studies^32^, ^33^, ^34^, ^35^ with 1634 and 425 735 individuals, respectively. A reference list of included and excluded reports can be found in Table S4. Excluded trials with reasons for exclusion are listed in Table S5.

### 3.2 Study characteristics

For study characteristics, see Table 1. One study included women with a singleton pregnancy and a birth after 24 weeks.^33^ The other studies excluded high‐risk women to varying degrees, based on the following conditions: previous PTB, sPTB, second‐trimester abortion, or prior cervical surgery. Women were screened with vaginal ultrasound between 16–20 weeks and 23–24 weeks and subsequently treated until 36 or 37 weeks if the cervix was shortened. The studies included different cut‐offs for cervical length to be subject to treatment; the most frequently occurring was ≤25 mm, two studies used <25 mm and ≤20 mm, respectively. Rates of short cervix were 0.9%–6.6%. The treatment rate of vaginal progesterone was 1.4% and 2.2% in the RCTs and 0.2%–6.6% in the cohort studies. No study used oral progesterone. One study used progesterone treatment exclusively.^31^ Adherence to treatment was only noted in Melchor et al.^34^

**TABLE 1:** Characteristics of included studies.

Author year country	Study design, study period	Timing of TVU, cervical length cut‐off	Exclusion criteria	Interventions for prevention of PTB	Concomitant intervention	Number of participants	Outcome variables Primary outcome in bold text
Mishra 2018 India, one tertiary center	RCT, July 2014 to Dec 2015	16–24 w, ≤25 mm	Prior second‐trimester abortion or singleton sPTB (>16 to <37 w), >2 first‐trimester abortions, multifetal pregnancy, fetal malformations, medical disorders	*Intervention:* vaginal progesterone 200 mg daily until 37 w *Control:* routine care	*Intervention + Control:* If PTL or PPROM: iv fluid, tocolysis, Betamethasone. If PPROM, the addition of antibiotics and discontinuation of vaginal progesterone	Intervention: 150 (screened) Control: 150 (not screened)	**sPTB < 37 w** 32 + 0–36 + 6 w, 28 + 0–31 + 6 w, <28 w. Birth weight Composite neonatal adverse outcome (NNM, RDS, IVH, jaundice, or NICU admission). NNM, RDS, IVH, jaundice, and NICU admission were also reported.
Saccone 2024 Italy, 2 centers	RCT, July 2018 to Dec 2022	18 + 0–23 + 6 w, ≤25 mm	Multifetal pregnancy, or previous sPTB. At randomization: PPROM, bleeding, symptoms of PTL, major malformations, cerclage or pessary in situ	*≤25 mm:* Intervention: Vaginal progesterone 200 mg daily and pessary until 36 w *25.1 to 29.9 mm:* TVU <1 week, if ≤25 mm, 200 mg progesterone daily and pessary, if >25 mm, no further exam. *Control:* Routine care	*Cervical length ≤ 25 mm:* I: Pessary until 36 w *If cervical length ≤5 mm:* speculum exam, and if dilation ≥15 mm or visible membranes: physical examination‐indicated cerclage and progesterone	Intervention: 675 (screened) Control: 659 (not screened)	**PTB < 37 w** PTB <34, <32, <30, <28, <24 w sPTB <37, 34, 32, 30, 28, 24 w Birth weight, NICU admission, NNM <28 d, stillbirth, NNM Composite neonatal adverse Outcome (NNM, IVH ≥ grade 3, NEC, RDS, BPD, proven sepsis.) IVH ≥grade 3, NEC, RDS, BPD, and proven sepsis were also reported
Figarella 2023, France multi‐center, 41 maternity centers	Cohort before‐after, Before (period A) Jan 1, 2012 to Dec 3, 2014, After (period B) May 1, 2015, to April 30, 2018	At the time of the second‐trimester anatomy scan, ≤25 mm	Multifetal pregnancy, delivery <24 w	Vaginal progesterone 200 mg daily, cerclage or pessary	–	*Period A (Control)* 171 079 (not screened group), 49 504, (28.9%) were screened. *Period B (I)* 165 524 (screened group) 87 546 (52.9%) were screened	**PTB < 37 w** induced PTB, length of hospital stay for PTL, no of hospital admissions NICU admissions, neonatal resuscitation, stillbirth
Melchor Corcostegui, 2023, Spain, single center	Cohort before‐after, Jan 1, 2011, to Dec 31, 2011 (not screened) and Jan 1, 2018, to Dec 31, 2018 (screened)	19 + 0–22 + 6 w, <25 mm	Previous sPTB	Vaginal progesterone 200 mg daily until 36 + 6 w	Cerclage if cervical length < 10 mm or progressive cervical shortening in women with cervical length <25 mm	Women included in analysis/ all women presenting with threatening PTL: Intervention: 483/628 (screened) Control: 410/482 (not screened)	Threatening PTL, admission for PTL, length of hospital stay, sPTB <37, <35, <34, <32, <28 w
Son, 2016 United States, single center	Cohort before‐after, Jan 2007 to Jan 2014	18–24 w, ≤25 mm	<18 years, multifetal pregnancy, previous PTB <37 w, spontaneous miscarriage at <20 w or if terminating pregnancy <24 w	≤20 mm: vaginal progesterone 200 mg daily >20‐ ≤ 25 mm, follow up at <24 w	≤25 mm, also digital cervical examination and cerclage if cervical dilation <24 w	Intervention: 17609 (screened) Control: 46 598 (not screened)	**PTB < 37 w** PTB <34, <32 w sPTB <37, <34, <32 w
Souka 2024 Greece, multi‐center 2 private centers	Cohort propensity score matched, Jan 2006 to Dec 2015	20–24 w, ≤15 mm	Previous sPTB, second‐trimester abortion, history of cervical surgery or congenital uterine malformations, singletons from embryo reduction, intrauterine death of one twin	Vaginal progesterone 200 mg daily or cerclage or pessary, at the discretion of the obstetrician	Cerclage or pessary added if persistent cervical shortening, despite progesterone	Intervention: 3103 (screened) Control: 3103 (not screened) Original sample 10 133 (6913 screened, 3220 not screened)	sPTB between 24 and 31 + 6 w sPTB <32 w or spontaneous miscarriage between 20 and 23 w

*Note*: Bold outcome values for Mishra: RR/OR not presented, *p*‐value 0.43; Saccone: RR 0.86 (0.59–1.25), *p*‐value 0.43; Figarella: aOR 0.92 (0.89–0.95), *p*‐ value <0.0001; Son: OR 0.88 (0.82–0.94), aOR 0.82 (0.76–0.88), *p*‐value not presented.

Abbreviations: BPD, bronchopulmonary dysplasia; IVH, intraventricular hemorrhage; NEC, necrotizing enterocolitis; NICU, neonatal intensive care unit; NNM, neonatal mortality; PPROM, preterm prelabor rupture of the membranes; PTB, preterm birth; PTL, preterm labor; RCT, randomized controlled study; RDS, respiratory distress syndrome; sPTB, spontaneous preterm birth; TVU, transvaginal ultrasound; w, weeks.

### 3.3 Study quality, including risk of bias assessment

**FIGURE 2:**
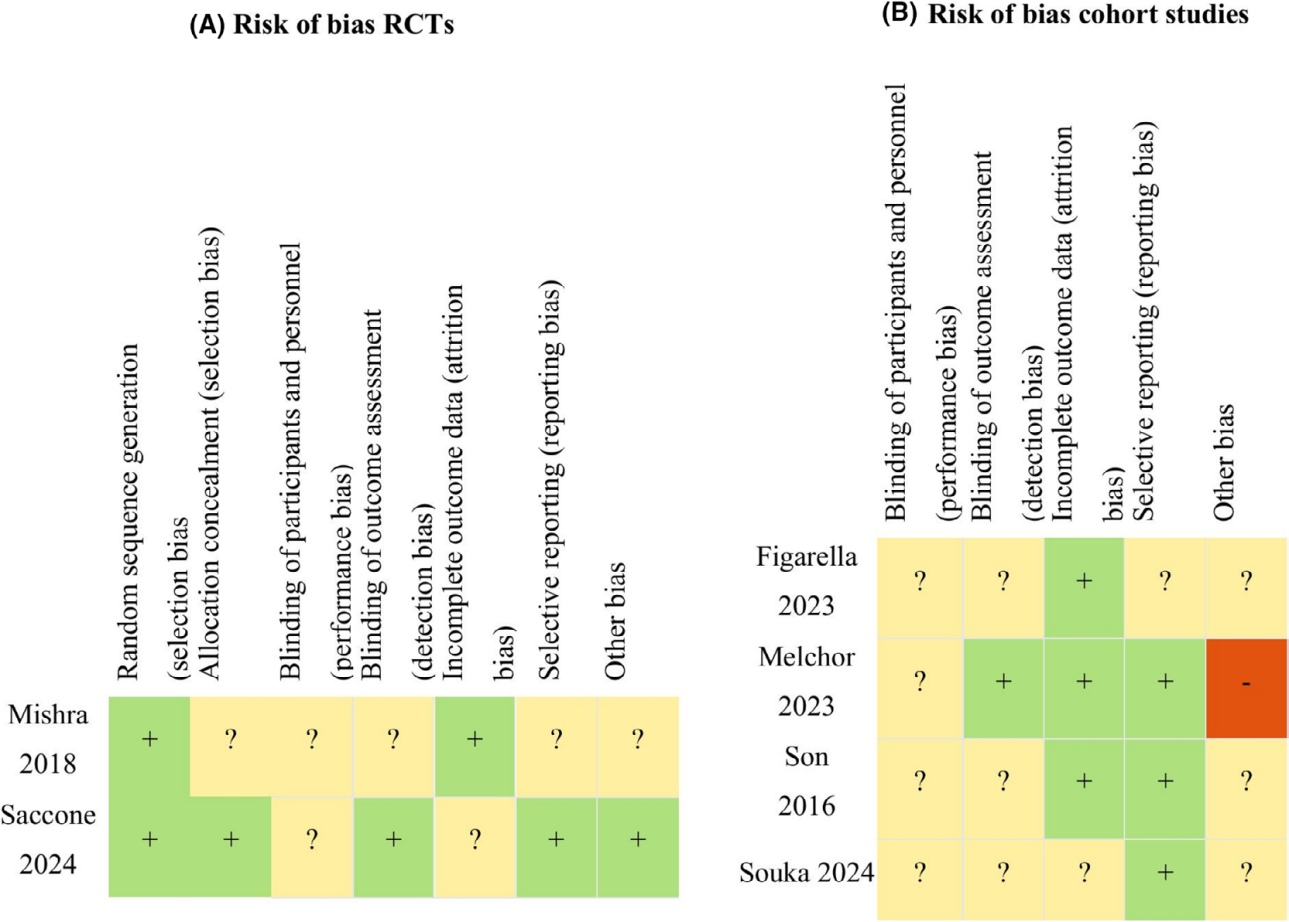
(A, B) Details of risk of bias for each study, RCTs, and cohort studies, respectively. Green = no problems, yellow = some problems, red = major problems.

For a summary of critical appraisal for each study, see Tables 2 and S6 for more details of critical appraisal for each study. Details of risk of bias for RCTs and cohort studies are shown in Figure 2A,B, respectively. One of the RCTs^31^ had some problems related to directness, as the clinical setting and selection process were inadequately described, and the study population included an unreasonably high proportion of high‐risk patients. Both RCTs demonstrated some risk of bias (e.g., lack of blinded assessment, missing information) and serious imprecision due to a low number of participants, few events, and wide CI.

**TABLE 2:** Summary of critical appraisal for each study.

Author, year	Study design	Directness	Risk of bias	Precision
Mishra, 2018	RCT	?/−	?	–
Saccone, 2024	RCT	+	?	–
Figarella, 2023	Cohort (before‐after)	?	?/−	+
Melchor, 2023	Cohort (before‐after)	–	–	?
Son, 2016	Cohort (before‐after)	+	?	+
Souka, 2024	Cohort (propensity score matched)	?	–	–

*Note*: Summary of critical appraisal for all included studies in the domains of directness, risk of bias, and precision. Green = no problems, yellow = some problems, red = major problems.

Abbreviation: RCT, randomized controlled study.

Three of the cohort studies showed some or major issues with directness. In Souka et al.,^32^ the study population and intervention were not clearly defined. In Figarella et al.,^33^ 28.9% of control group participants were nonetheless screened, and the number of patients receiving progesterone was unclear. Melchor et al.^34^ had major problems, as no baseline characteristics of the study population were reported. All cohort studies exhibited some or major risk of bias (e.g., lack of blinded assessment, missing data). Major selection bias was found in two cohort studies: Melchor et al.^34^ did not report potential confounders, and Souka et al.^32^ had a high risk of selection bias in the control group, as propensity score matching excluded 3810 of 6913 eligible participants. Two cohort studies demonstrated problems with imprecision. Souka et al.^32^ had major issues due to the absence of a power calculation and few events, while Melchor et al.^34^ had some concerns, as there was no power calculation and the primary outcome was onset of preterm labor rather than PTB.

### 3.4 Effect of intervention

**FIGURE 3:**
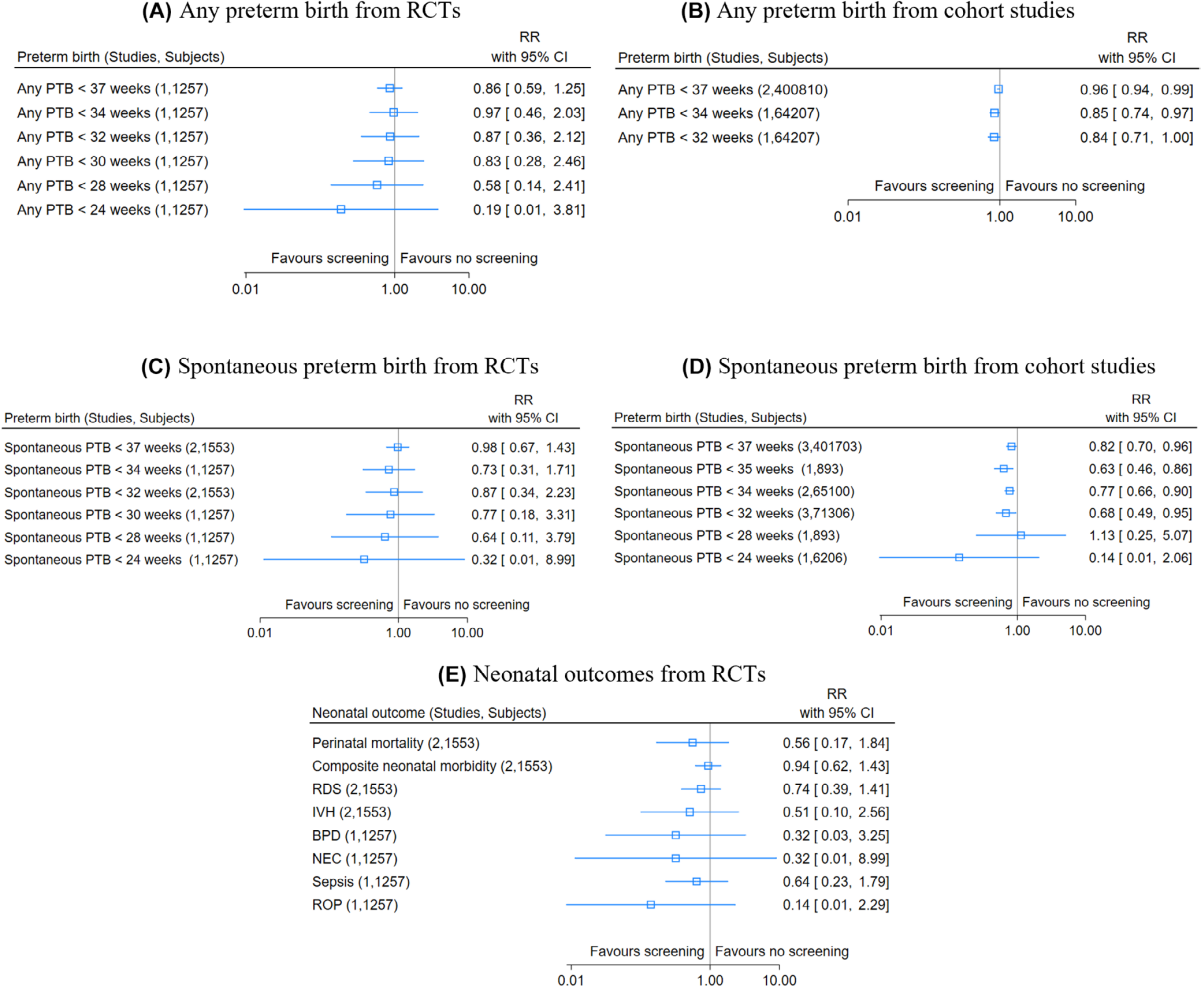
(A–E) Summary graph of all outcomes. Results are presented separately for RCTs and cohort studies. Pooled estimates are from meta‐analyses when possible.

Complete outcome tables are presented in Tables S7A–D. A summary of the results per outcome and its associated certainty of evidence is presented in Table 3. Details of the results of the GRADE assessment for RCTs and cohort studies separately for each outcome are shown in Table 4. The GRADE assessment resulted in the same rating for both RCTs and cohort studies and is therefore reported together for each outcome. Figure 3A–E summarize the crude estimates and, where available, the pooled estimates from meta‐analyses of studies reporting any PTB or sPTB (any PTB at <37, <34, <32, <30, <28, and <24 weeks, and sPTB at <37, <35, <34, <32, <30, <28, and <24 weeks) and related neonatal outcomes. Forest plots per outcome are presented in Figure S8A–R.

**TABLE 3:** Summary of findings in randomized controlled trials and cohort studies: Transvaginal ultrasound screening of cervical length versus no screening.

Outcomes	Number of RCTs and cohort studies (patients)	Absolute effect* screening vs no screening *n*/*N* (%)	Relative effect RR (95% CI) *p* value	Certainty of evidence GRADE**
Any PTB < 37 weeks	1 (1257) 2 (400810)	48/639 (7.5) vs. 54/618 (8.7) 10 326/183133 (5.6) vs. 13 044/217677 (6.0)	0.86 (0.59–1.25) n.s. **0.96 (0.93–0.98)** ***p* = 0.0005** **aOR** **0.87 (0.78–0.98)** ***p* = 0.017**	Very low (GRADE ⊕OOO)^a^
Any PTB < 34 weeks	1 (1257) 1 (64207)	14/639 (2.2) vs. 14/618 (2.3) 291/17609 (1.7) vs. 907/46598 (2.0)	0.97 (0.46–2.01) n.s. **0.85 (0.74–0.97)** **p = 0.01**	Very low (GRADE ⊕OOO)^b^
Any PTB < 32 weeks	1 (1257) 1 (64207)	9/639 (1.4) vs. 10/618 (1.6) 168/17609 (1.0) vs. 532/46598 (1.1)	0.87 (0.36–2.13) n.s. **0.84 (0.70–0.99) *p* = 0.04**	Very low (GRADE ⊕OOO)^b^
Any PTB < 30 weeks	1 (1257) –	6/639 (0.9) vs. 7/618 (1.1)	0.83 (0.28–2.45) n.s.	Very low (GRADE ⊕OOO)^b^
Any PTB < 28 weeks	1 (1257) NA	3/639 (0.5) vs. 5/618 (0.8)	0.58 (0.14–2.42) n.s.	Very low (GRADE ⊕OOO)^b^
Any PTB < 24 weeks	1 (1257) –	0/639 (0.0) vs. 2/618 (0.3)	0.19 (0.01–4.02) n.s.	Very low (GRADE ⊕OOO)^b^
Spontaneous PTB < 37 weeks	2 (1553) 3 (401703)	51/786 (6.5) vs. 51/767 (6.7) 3754/183616 (2.0) vs. 5625/218087 (2.6)	0.98 (0.67–1.42) n.s. **0.82 (0.70–0.96)** ***p* = 0.02**	Very low (GRADE ⊕OOO)^a^
Spontaneous PTB < 35 weeks	NA 1 (893)	59/483 (12.2) vs. 90/410 (19.5)	**0.63 (0.46–0.85)** ***p* = 0.003**	Very low (GRADE ⊕OOO)^c^
Spontaneous PTB < 34 weeks	1 (1257) 2 (65100)	9/639 (1.4) vs. 12/618 (1.9) 211/18092 (1.2) vs. 638/47008 (1.4)	0.73 (0.31–1.71) n.s. **0.77 (0.66–0.90)** ***p* = 0.001**	Very low (GRADE ⊕OOO)^a^
Spontaneous PTB < 32 weeks	2 (1553) 3 (71306)	8/786 (1.0) vs. 9/767 (1.2) 116/21195 (0.6) vs. 366/50111 (0.7)	0.87 (0.34–2.23) n.s. 0.68 (0.49–0.95) ***p* = 0.03**	Very low (GRADE ⊕OOO)^a^
Spontaneous PTB < 30 weeks	1 (1257) NA	4/639 (0.6) vs. 5/618 (0.8)	0.77 (0.21–2.87) n.s.	Very low (GRADE ⊕OOO)^b^
Spontaneous PTB < 28 weeks	1 (1257) 1 (893)	2/639 (0.3) vs. 3/618 (0.5) 4/483 (0.8) vs. 3/410 (0.7)	0.64 (0.11–3.85) n.s. 1.13 (0.25–5.03) n.s.	Very low (GRADE ⊕OOO)^d^
Spontaneous PTB < 24 weeks	1 (1257) 1 (6206)	0/639 (0.0) vs. 1/618 (0.2) 0/3103 (0.0) vs. 2/3103 (0.1)	0.32 (0.01–7.90) n.s. 0.14 (0.01–2.16) n.s.	Very low (GRADE ⊕OOO)^e^
Spontaneous PTB 32–37 weeks	1 (296) NA	13/147 (8.8) vs. 11/149 (7.4)	1.20 (0.55–2.59) n.s.	Very low (GRADE ⊕OOO)^a^
Perinatal mortality***	2 (1553) NA	4/786 (0.5) vs. 7/767 (0.9)	0.56 (0.17–1.84) n.s.	Very low (GRADE ⊕OOO)^a^
Composite neonatal morbidity****	2 (1553) NA	40/786 (5.1) vs. 42/767 (5.5)	0.94 (0.62–1.43) n.s.	Very low (GRADE ⊕OOO)^a^
Respiratory distress syndrome	2 (1553) NA	16/786 (2.0) vs. 21/767 (2.7)	0.74 (0.39–1.41) n.s.	Very low (GRADE ⊕OOO)^a^
Intra‐ventricular hemorrhage	2 (1553) NA	2/786 (0.3) vs. 4/767 (0.5)	0.51 (0.10–2.52) n.s.	Very low (GRADE ⊕OOO)^f^
Certainty of evidence				
High certainty: ⊕⊕⊕⊕	We are very confident that the true effect lies close to that of the estimate of the effect			
Moderate certainty: ⊕⊕⊕O	We are moderately confident in the effect estimate: The true effect is likely to be close to the estimate of the effect, but there is a possibility that it is substantially different			
Low certainty: ⊕⊕OO	Confidence in the effect estimate is limited: The true effect may be substantially different from the estimate of the effect			
Very low certainty: ⊕OOO	We have very little confidence in the effect: The true effect is likely to be substantially different from the estimate of the effect			

*Note*: All included outcomes are presented with the total number of participants, absolute and relative effect, *p*‐value, and participants in intervention and control with percentages, together with associated certainty of evidence. Numbers in bold indicate that the difference is statistically significant.

Abbreviations: aOR, adjusted odds ratio; *n*, number; NA, not available; n.s., non‐significant; PTB, preterm birth; RCT, randomized controlled trial; RR, risk ratio.

^a^ Downgraded one level for some study limitations, some inconsistency, and some indirectness, two levels for very serious imprecision.

^b^ Downgraded one level for some study limitations and some inconsistency, two levels for very serious imprecision.

^c^ Downgraded one level for serious study limitations, one level for serious indirectness, and one level for serious imprecision.

^d^ Downgraded one level for serious study limitations and serious indirectness, two levels for very serious imprecision.

^e^ Downgraded one level for serious study limitations, two levels for very serious imprecision.

^f^ Downgraded one level for some study limitations and some indirectness, two levels for very serious imprecision.

* Absolute effects for event rates in the intervention group versus the control group, presented as the sum of all events/the total number of participants.

** The GRADE assessment resulted in the same rating for both RCTs and cohort studies and is therefore reported together for each outcome.

*** Perinatal mortality: intrauterine fetal death and neonatal mortality <7 or <28 days.

**** Neonatal morbidity: A composite outcome of neonatal morbidity (at least one of bronchopulmonary dysplasia, severe intraventricular hemorrhage, necrotizing enterocolitis, confirmed sepsis, retinopathy of prematurity) with or without perinatal mortality.

**TABLE 4:** Results from GRADE assessments per outcome.^a^

Outcome	Risk of bias		Consistency		Directness		Precision	
	RCT	Cohort	RCT	Cohort	RCT	Cohort	RCT	Cohort
Any PTB < 37 weeks								
Any PTB < 34 weeks								
Any PTB < 32 weeks								
Any PTB < 30 weeks		–		–		–		–
Any PTB < 28 weeks		–		–		–		–
Any PTB < 24 weeks		–		–		–		–
Spontaneous PTB < 37 weeks								
Spontaneous PTB < 35 weeks	–		–		–		–	
Spontaneous PTB < 34 weeks								
Spontaneous PTB < 32 weeks								
Spontaneous PTB < 30 weeks		–		–		–		–
Spontaneous PTB < 28 weeks								
Spontaneous PTB < 24 weeks								
Spontaneous PTB < 32–37 weeks		–		–		–		–
Perinatal mortality		–		–		–		–
Composite neonatal morbidity		–		–		–		–
Respiratory distress syndrome		–		–		–		–
Intraventricular hemorrhage		–		–		–		–

^a^ Assessment for risk of bias, consistency, directness, and precision for all outcomes labeled with green, yellow, and red for no, some, and major problems, respectively.

### 3.5 Any PTB

Any PTB was reported in one RCT^30^ and two cohort studies,^33^, ^35^ all with some risk of bias. The RCT (*n* = 1257) did not show any reduction in any PTB for <37, >34, <32, <30, <28, and <24 weeks. Pooling the results of the two cohort studies in meta‐analyses showed a reduction in any PTB < 37 weeks: 5.6% (103 276/183133) in the screened group versus 6.0% (13 044/217677) in the unscreened group (aOR 0.87 [95% CI 0.78 to 0.98], *p*‐value 0.017) with number needed to screen to benefit 263 (95% CI 190 to 424) (very low certainty of evidence). One cohort study^36^ reported a reduction in any PTB <34 weeks: 1.7% (291/17609) in the screened group versus 2.0% (907/46598) in the unscreened group (RR 0.85 [95% CI 0.74 to 0.97], *p*‐value 0.01) and for <32 weeks 1.0% (168/17609) in the screened group versus 1.1% (532/46598) in the unscreened group (RR 0.84 [95% CI 0.70 to 0.99], *p*‐value 0.04) (very low certainty of evidence).

### 3.6 Spontaneous PTB

Spontaneous PTB was reported in all studies included in this review. The RCTs had some risk of bias, and the cohort studies had some risk of bias for all outcomes except for sPTB <35, <28, and <24 weeks, which had a major risk of bias. Meta‐analyses of the RCTs (*n* = 1553) showed no reduction in sPTB for any prespecified week. Meta‐analyses of three cohort^33^, ^34^, ^35^ studies showed a reduction in sPTB < 37 weeks: 2.0% (3754/183616) in the screened group versus 2.6% (5625/218087) in the unscreened group (RR 0.82 [95% CI 0.70 to 0.96], p‐value 0.02) with number needed to screen to benefit 186 (95% CI 159 to 227) (very low, *p*‐value 0.0027) and a reduction in sPTB < 34 weeks^34^, ^35^: 1.2% (211/18092) in the screened group versus 1.4% (638/47008) in the unscreened group (RR 0.77 [95% CI 0.66 to 0.90], *p*‐value 0.001) (very low certainty of evidence). Similar results were assessed for sPTB < 32 weeks^32^, ^34^, ^35^: 0.6% (116/21195) in the screened group versus 0.7% (366/50111) in the unscreened group (RR 0.68 [95% CI 0.49 to 0.95], *p*‐value 0.03) (very low certainty of evidence). In one cohort study, no significant reduction was observed in sPTB before 35 or 28 weeks (Melchor et al.^34^), and similarly, Souka et al.^32^ reported no reduction in sPTB before 24 weeks.

### 3.7 Neonatal outcomes

Neonatal outcomes were only reported in the two RCTs (for specified neonatal outcomes, see Table 3, Figures 3E, and S8A–R). There was no reduction in perinatal mortality or serious neonatal morbidity.

### 3.8 Subgroup analyses

Planned subgroup analyses were not executed due to a lack of data.

## 4 DISCUSSION

The main finding of this SR is that it remains uncertain whether universal cervical length screening using transvaginal ultrasound, followed by vaginal progesterone therapy, with or without additional interventions for women identified with a short cervix, reduces the risk of PTB or adverse neonatal outcomes compared with no screening. This conclusion applies to all assessed outcomes and is primarily based on meta‐analyses of RCTs, given the high risk of bias in the included cohort studies.

Specifically, no clear differences in any PTB, sPTB, and neonatal outcomes were shown in the RCTs. In general, RCTs are less susceptible to bias compared to observational studies, and therefore, generally acquire a higher certainty of evidence.^37^ However, a major limitation of the RCTs included in this SR was their small sample sizes (300 and 1334 participants, respectively), thus reducing the certainty of evidence. Both Saccone et al.^30^ and Mishra et al.^31^ based their sample size calculations on optimistic assumptions regarding the expected reduction in sPTB. Saccone et al. assumed a 50% relative reduction in sPTB (from 6.7% in the control group), while Mishra et al. assumed a reduction from 15% to 5% (an absolute reduction of 10 percentage points, corresponding to a 67% relative reduction). The observed reduction was considerably smaller, from 8.7% to 7.5% in Saccone et al.,^30^ and there was an increase from 8.1% to 10.2% in Mishra et al.^31^ Based on the effect size reported in Saccone et al.,^30^ the trial would have needed 16 228 participants to detect a statistically significant difference between groups.

In contrast, we found a reduction of PTB in the meta‐analyses of the included cohort studies that might indicate that universal screening could be beneficial, especially for sPTB <37, <24, and <32 weeks, with a RR between 0.68 and 0.82, corresponding to a number needed to screen of 263 to benefit from any PTB < 37 weeks and 186 for sPTB < 37 weeks. However, most of the included cohort studies did not report baseline characteristics, and only two provided results from adjusted analyses. Furthermore, the magnitude of the observed effect was not large enough to justify a higher level of certainty in any of the meta‐analyses.^27^ Accordingly, the strength of evidence from the cohort studies was therefore rated as very low.

A recent SR on the same clinical question by Hessami et al.^38^ came to a different conclusion from ours. They concluded that universal cervical length screening in women with a singleton pregnancy and no prior PTB was associated with a statistically significant reduction in PTB before 37 weeks compared with no screening (OR 0.88; 95% CI 0.79 to 0.97). However, they found no significant association between screening and a reduction in sPTB before 37 weeks, when both high‐ and low‐risk women were included. Notably, Hessami et al.^38^ included three studies that were excluded from the present SR: one used abdominal ultrasound for screening, and two used women who declined screening as the control group. Additionally, the RCT by Saccone et al.^30^ was not included, as it had not yet been published. An important methodological difference was that Hessami et al.^38^ included both RCTs and cohort studies in the same meta‐analyses. Consequently, the overall result from their meta‐analyses is largely driven by observational data, as the only included RCT was very small. Given the clinical and methodological heterogeneity, we consider that RCTs and cohort studies should be analyzed separately.^27^

In an editorial in the *American Journal of Obstetrics & Gynecology MFM*, there was a call for implementing universal cervical length screening,^18^ and this approach was subsequently endorsed by the International Society of Ultrasound in Obstetrics and Gynecology (ISUOG) in 2022.^39^ However, the remaining majority of national and international obstetric and gynecological organizations continued to recommend cervical length screening primarily for women at high risk of PTB.^40^, ^41^, ^42^ This recommendation is in line with the results of our SR.

In some settings, clinical adoption may have preceded the availability of high‐certainty evidence, likely reflecting both the strong desire to prevent PTB and the perception of low intervention‐related risks. Nevertheless, experience from obstetrics has shown that biologically plausible and apparently low‐risk interventions may ultimately prove ineffective, or even harmful, when evaluated in adequately powered RCTs.^43^, ^44^ Continued critical appraisal of the evidence base is therefore essential.

The major strength of this SR is the robust methodology, including predefined eligibility criteria, structured risk‐of‐bias assessment, and separate analysis of RCTs and cohort studies. The authors were selected based on specific areas of expertise needed for the assessment. The included studies were all published during the last decade, making the results up to date with current knowledge. In the retrospective cohort studies, a large number of participants were included, yielding meta‐analyses with up to 401 703 individuals. In addition, there appears to be consensus on key aspects of management, including the definition of a short cervix as ≤/<25 mm in all but one study, and the use of progesterone as the first‐line treatment for a short cervix.

A major limitation of this review is the small number of eligible studies, six in total, of which only two were RCTs. Furthermore, most studies excluded women at high risk of PTB, particularly those with a prior history of PTB. In this review, as prespecified in our PICO framework, “universal screening” refers to offering cervical length assessment to a general obstetric population rather than restricting screening according to baseline risk status. However, the exclusion of high‐risk women may limit generalizability.

Another limitation relates to the intervention itself. Few participants received progesterone treatment: only 17 women (1.4% and 2.2%, respectively) in the RCTs received progesterone, and between 0.2% and 6.6% in the cohort studies. In addition, all studies except one,^31^ combined treatments in different ways. In Saccone et al.,^30^ all women with a short cervix were offered a pessary insertion in addition to progesterone treatment, and most studies added cerclage if progressive cervical shortening or dilation. This approach likely reflects real‐world clinical practice in many settings but limits the generalizability of the findings concerning the effect of progesterone. Finally, five of the six studies included in this SR were performed in Italy, the United States, France, Spain, and Greece. Accordingly, the findings are most likely applicable to populations in high‐income countries, resulting in limited generalizability for low‐and middle‐income countries.

In conclusion, high‐certainty evidence supporting universal cervical length screening and progesterone treatment to prevent PTB is lacking. The RCTs showed no significant reduction in PTB, whereas the cohort studies showed a reduction, but with very low certainty of evidence. However, due to concerns related to indirectness and risk of bias in these studies, it cannot be determined with certainty that the screening itself causes this reduction. The RCTs, on the other hand, were underpowered, and it cannot be ruled out that significantly larger RCTs might demonstrate a clinically significant effect.

From a health system perspective, implementation of universal cervical length screening would be most feasible in settings with publicly funded and standardized antenatal care, such as the Nordic countries, where screening could be integrated into the routine second‐trimester ultrasound examination. This approach would likely ensure high coverage while minimizing additional attendance costs, but it requires access to transvaginal ultrasound, structured training, and ongoing quality assurance to ensure measurement reproducibility. In a Swedish context, the practical requirements and cost‐effectiveness of such a strategy have recently been evaluated using a decision‐analytic model.^7^ That analysis suggested that universal screening followed by vaginal progesterone treatment for women with a short cervix could reduce sPTB and be cost‐effective compared with no screening. However, these estimates are model‐based and depend on assumptions regarding treatment effectiveness, which remain uncertain given the limited high‐certainty evidence from RCTs.

Although widespread adoption and the perceived low‐risk profile of screening and treatment may reduce enthusiasm for further randomization in some settings, substantial scientific uncertainty remains regarding the true population‐level impact.

From a public health and funding perspective, large‐scale implementation of a screening strategy requires robust evidence of clinically meaningful and cost‐effective benefits. When interventions are introduced at a population level, a high level of evidence is warranted.

An alternative to a traditional individually randomized parallel‐group RCT could be a national stepped‐wedge cluster RCT,^45^ introducing cervical length screening sequentially across regions or clinics within routine care. Such a design may facilitate more feasible recruitment and implementation while generating high‐quality evidence within real‐world clinical practice. An individual participant data meta‐analysis could provide additional insight through standardized adjustment and subgroup exploration. However, given the limited number and size of existing RCTs, adequately powered prospective trials remain essential. Further trials may also include an evaluation of the timing of screening and other cervical length thresholds for progesterone treatment to improve the effectiveness of a universal cervical length screening program.

## 5 CONCLUSION

Given the very low certainty of the available evidence, it remains unclear whether universal cervical length screening, followed by progesterone treatment in women identified with a short cervix, reduces the incidence of PTB or improves neonatal outcomes compared with no screening. More adequately powered trials are needed before a definitive recommendation can be made.

## AUTHOR CONTRIBUTIONS

Mira Zethelius: Conceptualization, data curation, formal analysis, investigation, validation, visualization, and writing‐original draft. Lina Bergman, Bo Jacobsson, and Pihla Kuusela: Conceptualization, data curation, formal analysis, investigation, validation, and writing‐review and editing. Ann‐Catrin Ekelund and Ann Liljegren: Conceptualization, data curation, investigation, methodology, and validation. Cecilie Hongslo Vala, Björn Lindkvist, and Petteri Sjögren: Conceptualization, data curation, formal analysis, investigation, methodology, project administration, software, validation, and writing‐review and editing. Max Petzold: Conceptualization, data curation, formal analysis, investigation, methodology, software, and validation. Ulla‐Britt Wennerholm and Tove Wikström: Conceptualization, data curation, formal analysis, investigation, supervision, validation, and writing‐review and editing.

## FUNDING INFORMATION

The authors did not receive any specific funding for this work.

## CONFLICT OF INTEREST STATEMENT

BJ declares having led a working group within FIGO (International Federation of Gynecology and Obstetrics) on PTB and is now the FIGO Division Director of Maternal and Newborn Health. LB declares membership in the International Federation of Gynecology and Obstetrics (FIGO) on long‐term maternal health within the division of maternal and newborn health. ACE, AL, BL, CHV, MP, MZ, PK, PS, TW, and UBW have no conflicts of interest related to the content of this review.

## Supporting information


**Figure S8.** A‐R Forest plots for all outcomes.

**Table S1.** PRISMA checklist.

**Table S2.** Search strategy.

**Table S3.** PICO.

**Table S4.** Reference lists of included and excluded reports.

**Table S5.** Excluded articles with reasons for exclusion.

**Table S6.** Specification of aspects important for quality assessment.

**Table S7.** Outcome tables A‐D.

## Data Availability

All relevant data are within the manuscript and its Supporting Information files.
