# Supplementary material for: Can universal cervical length screening with vaginal progesterone for a short cervix reduce preterm birth? A systematic review and meta‐analyses
Source: Acta Obstet Gynecol Scand. 2026 May 20;105(8):1420–34. doi: 10.1111/aogs.70253 (PMC13356482; doi:10.1111/aogs.70253)

**Figures S8. A-R.** Forest plots for all outcomes

**Any PTB**

**A1:** Any preterm birth <37 weeks from one RCT. Risk ratio for screening versus no screening.


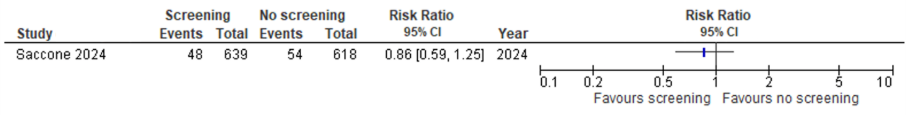


**A2:** Any preterm birth <37 weeks from cohort studies. Risk ratio for screening versus no screening.


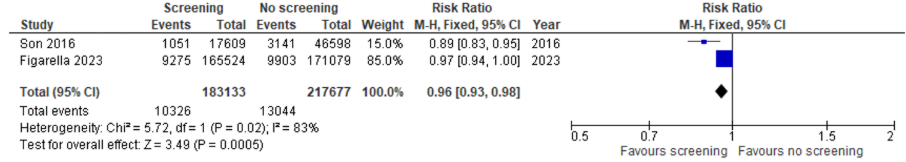


**A3:** Any PTB before 37+0 weeks from cohort studies. AOR* for screening compared with no screening**.**


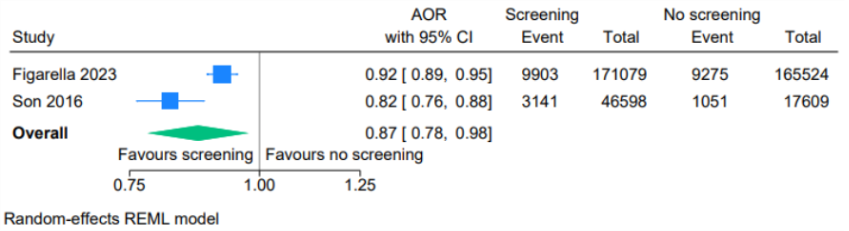


**B1:** Any preterm birth <34 weeks from one RCT. Risk ratio for screening versus no screening.


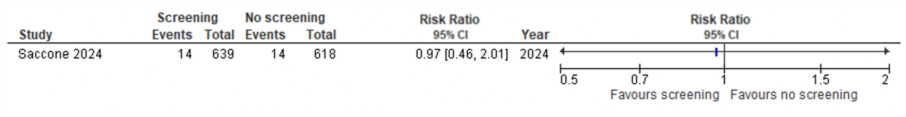


**B2:** Any preterm birth <34 weeks from one cohort study. Risk ratio for screening versus no screening.


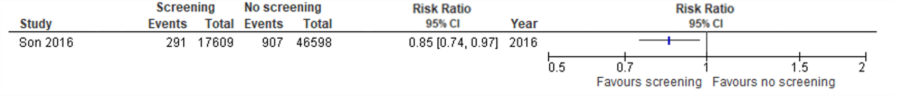


**C1:** Any preterm birth <32 weeks from one RCT. Risk ratio for screening versus no screening.


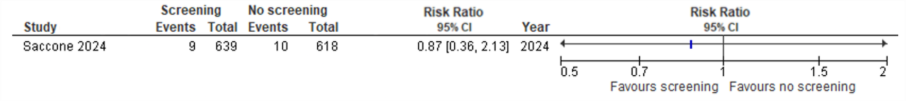


**C2:** Any preterm birth <32 weeks from one cohort study. Risk ratio for screening versus no screening.
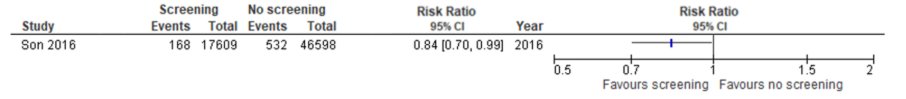


**D:**Any preterm birth <30 weeks from one RCT. Risk ratio for screening versus no screening.


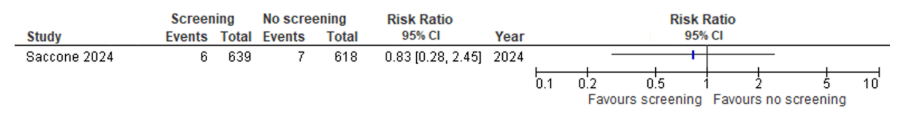


**E:** Any preterm birth <28 weeks from one RCT. Risk ratio for screening versus no screening.


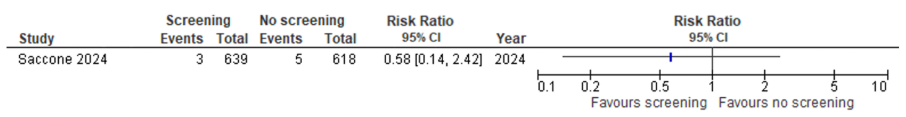


**F:** Any preterm birth <24 weeks from one RCT. Peto odds ratio for screening versus no screening.


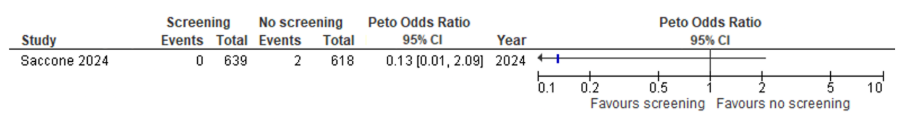


**Spontaneous PTB**

**G1:** Spontaneous preterm birth <37 weeks from RCTs. Risk ratio for screening versus no screening.


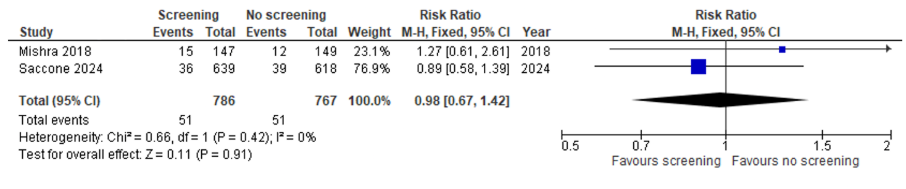


**G2:** Spontaneous preterm birth <37 weeks from cohort studies. Risk ratio for screening versus no screening.


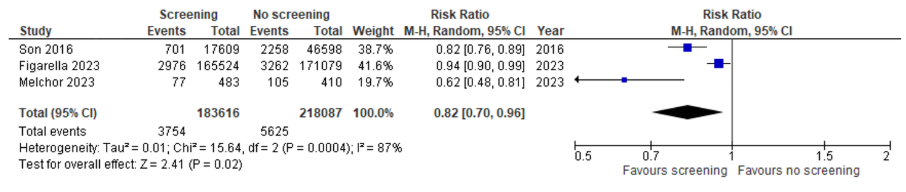


**H:** Spontaneous preterm birth between 32+0 and 36+6 weeks from one RCT. Risk ratio for screening versus no screening.


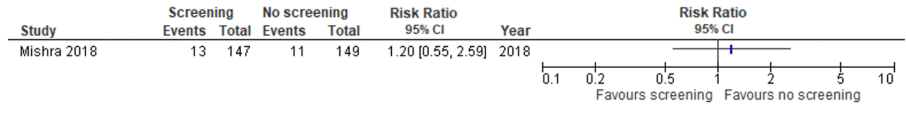


**I:** Spontaneous preterm birth <35 weeks from one cohort study. Risk ratio for screening versus no screening.
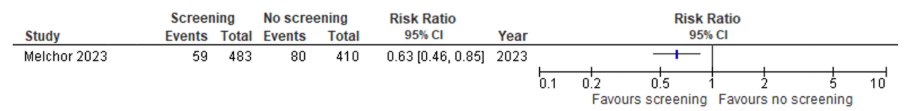


**J1:** Spontaneous preterm birth <34 weeks from one RCT. Risk ratio for screening versus no screening.
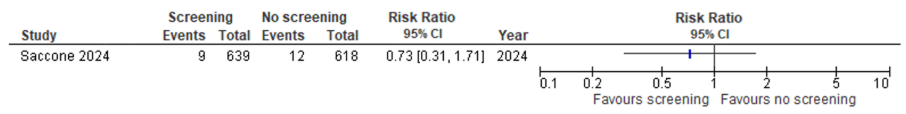


**J2:** Spontaneous preterm birth <34 weeks from cohort studies. Risk ratio for screening versus no screening.
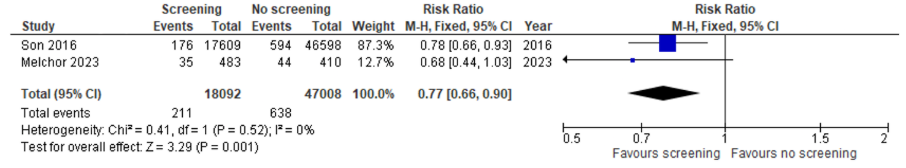


**K1:** Spontaneous preterm birth <32 weeks from RCTs. Risk ratio for screening versus no screening.


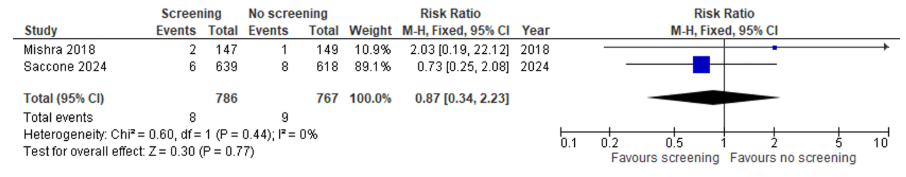


**K2:** Spontaneous preterm birth <32 weeks from cohort studies. Risk ratio for screening versus no screening.


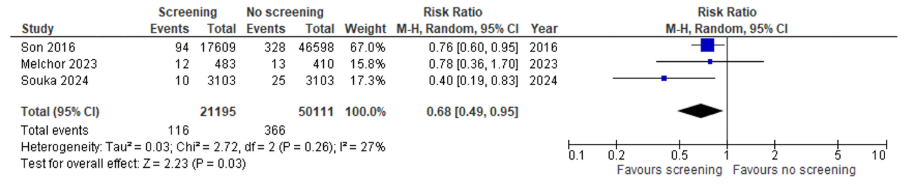


**L:** Spontaneous preterm birth <30 weeks from one RCT. Risk ratio for screening versus no screening.
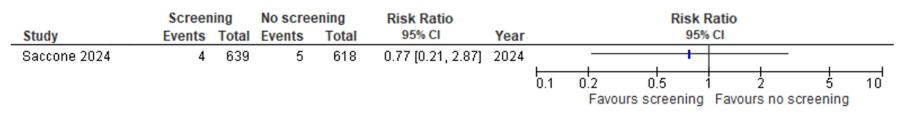


**M1:** Spontaneous preterm birth <28 weeks from one RCT. Risk ratio for screening versus no screening.
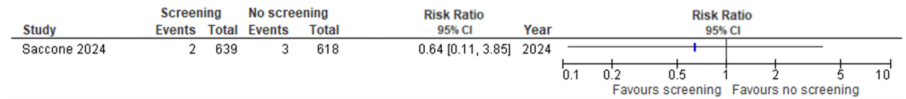


**M2:** Spontaneous preterm birth <28 weeks from one cohort study. Risk ratio for screening versus no screening.
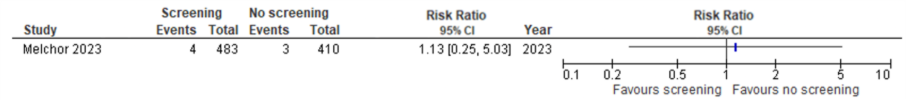


**N1:** Spontaneous preterm birth <24 weeks from one RCT. Peto odds ratio for screening versus no screening.
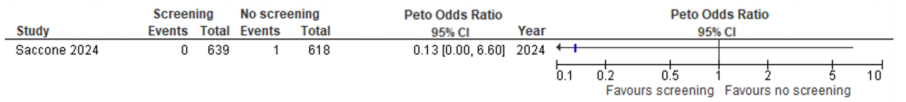


**N2:** Spontaneous preterm birth <24 weeks from one cohort study. Peto odds ratio for screening versus no screening.


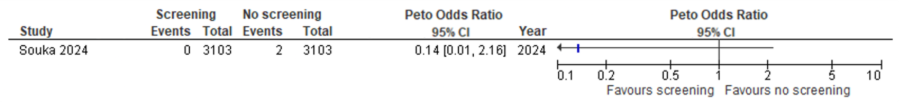


**Neonatal outcomes**

**O:** Perinatal mortality* from RCTs. Peto OR for screening vs no screening.


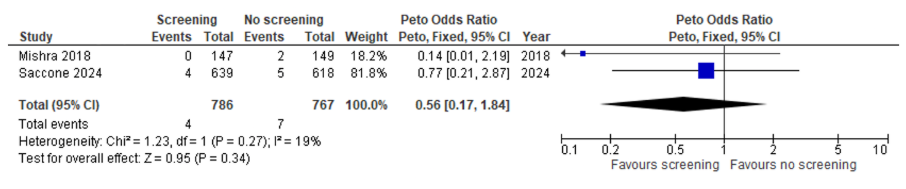


*Perinatal mortality: intrauterine fetal death and neonatal mortality <7 or <28 days

**P:** Composite adverse neonatal morbidity* from RCTs. Risk ratio for screening vs no screening.
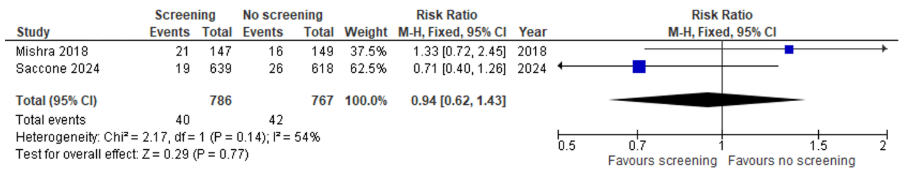


*Composite adverse neonatal outcome included at least one of bronchopulmonary dysplasia, severe intraventricular hemorrhage, necrotising enterocolitis, confirmed sepsis, retinopathy of prematurity with or without perinatal mortality

**Q:** Respiratory distress syndrome from RCTs. Risk ratio for screening vs no screening.


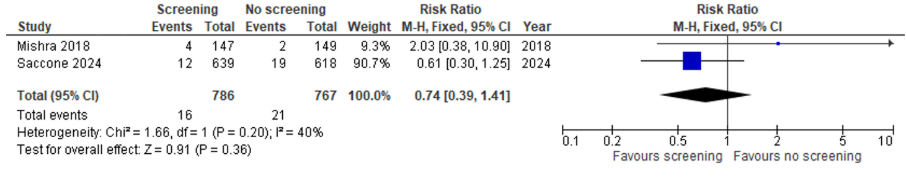


**R:** Intraventricular hemorrhage from RCTs. Peto odds ratio for screening vs no screening.


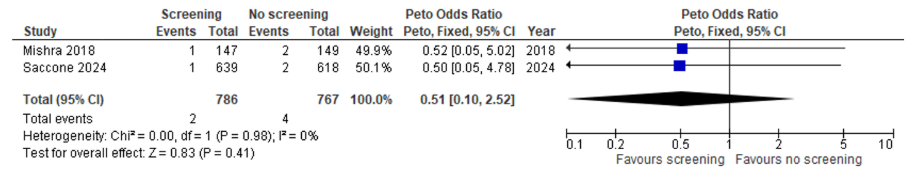

Supplement: Supplementary file 1 — Figure S8. A‐R Forest plots for all outcomes. [file AOGS-105-1420-s004.docx]
