# Supplementary material for: Can universal cervical length screening with vaginal progesterone for a short cervix reduce preterm birth? A systematic review and meta‐analyses
Source: Acta Obstet Gynecol Scand. 2026 May 20;105(8):1420–34. doi: 10.1111/aogs.70253 (PMC13356482; doi:10.1111/aogs.70253)
Supplement: Supplementary file 3 — Table S2. Search strategy. [file AOGS-105-1420-s008.docx]

**Table S2.** Search strategy

### Search strategy PROSPERO

**Database:** Medline ALL (OvidSP)
**Date:** 24 Oct 2024
**No. of results:** 1,686
**Search updated:** 2025-09-16, 79 results

| **Search** | **Query** | **Items found** |
| --- | --- | --- |
| 1 | exp Ultrasonography/ | 501868 |
| 2 | exp Mass Screening/ | 148254 |
| 3 | Cervix Uteri/di, dg | 2071 |
| 4 | (Echotomograph* or Echograph* or Sonograph* or Ultrasound* or Ultrason* or screening* or scan*).ab,kf,ti. | 1941569 |
| 5 | 1 or 2 or 3 or 4 | 2222554 |
| 6 | exp Cervical Length Measurement/ | 930 |
| 7 | (Cervi* adj4 (length* or measurement* or short* or assessment*)).ab,kf,ti. | 6964 |
| 8 | 6 or 7 | 7025 |
| 9 | Premature Birth/ | 23547 |
| 10 | exp Infant, Premature/ | 67969 |
| 11 | (Premature* or Pre-mature* or Preterm* or Pre-term*).ab,kf,ti. | 264757 |
| 12 | 9 or 10 or 11 | 280103 |
| 13 | 5 and 8 and 12 | 1882 |
| 14 | (comment or editorial or letter).pt. | 2288402 |
| 15 | 13 not 14 | 1800 |
| 16 | limit 15 to (Danish or English or Norwegian or Swedish) | 1686 |
| 17 | limit 16 to yr="1980 -Current" | **1686** |

**exp/** = term from the Medline controlled vocabulary, including terms found below this term in the MeSH hierarchy
**/** = term from the Medline controlled vocabulary, does not include terms found below this term in the MeSH hierarchy
**/di** = diagnosis a subheading to pinpoint a specific aspect of a subject heading concept.
**/dg** = diagnostic imaging a subheading to pinpoint a specific aspect of a subject heading concept.
**adj4** = next to each other, in any order, up to 3 word(s) in between
**.ab,kf,ti**. = abstract, author keyword and title
**.pt. =** publication type
***** at end of word = truncation of word for alternate endings

_________________________________________________________________________________________

**Database:** Embase 1974 to 2024 October 14 (OvidSP)
**Date:** 24 Oct 2024
**No. of results:** 2,065
**Search updated:** 2025-09-16, 104 results

| **#** | **Searches** | **Results** |
| --- | --- | --- |
| 1 | exp echography/ | 1081466 |
| 2 | exp mass screening/ | 336071 |
| 3 | uterine cervix/di [Diagnosis] | 2 |
| 4 | (Echotomograph* or Echograph* or Sonograph* or Ultrasound* or Ultrason* or screening* or scan*).ab,kf,ti. | 2725310 |
| 5 | 1 or 2 or 3 or 4 | 3493414 |
| 6 | cervical length measurement/ | 2791 |
| 7 | (Cervi* adj4 (length* or measurement* or short* or assessment*)).ab,kf,ti. | 10427 |
| 8 | 6 or 7 | 10789 |
| 9 | exp prematurity/ | 138090 |
| 10 | (Prematur* or Pre-matur* or Preterm* or Pre-term*).ab,kf,ti. | 361886 |
| 11 | 9 or 10 | 385929 |
| 12 | 5 and 8 and 11 | 3518 |
| 13 | limit 12 to (article or article in press or conference paper or note or "review") | 2250 |
| 14 | limit 13 to (Danish or English or Norwegian or Swedish) | 2073 |
| **15** | **limit 14 to yr="1980 -Current"** | **2065** |

**exp/** = term from the Embase controlled vocabulary, including terms found below this term in the Emtree hierarchy

**/** = term from the Embase controlled vocabulary, does not include terms found below this term in the Emtree hierarchy

**/di** = diagnosis a subheading to pinpoint a specific aspect of a subject heading concept.
**adj4** = next to each other, in any order, up to 3 word(s) in between
**.ab,kf,ti.** = abstract, author keyword and title
*****  at end of word = truncation of word for alternate endings

**Database:** The Cochrane Library
**Date:** 24 Oct 2024
**No of results:** 242 ref
**Search updated:** 2025-09-16, 0 results

*Cochrane reviews: 10*
*Cochrane protocols: 0*
*Trials: 232*

*Editorials: 0*
*Special collections: 0*
*Clinical answers: 0*

***Updated search 250916***
*Cochrane reviews: 10*
*Cochrane protocols: 0*
*Trials: 215*
*Editorials: 0*
*Special collections: 0*
*Clinical answers: 0*

| **#** | **Searches** | **Results** |
| --- | --- | --- |
| #1 | MeSH descriptor: [Ultrasonography] explode all trees | 19578 |
| #2 | MeSH descriptor: [Mass Screening] explode all trees | 6079 |
| #3 | MeSH descriptor: [Cervix Uteri] this term only and with qualifier(s): [diagnostic imaging - DG] | 132 |
| #4 | (Echotomograph* or Echograph* or Sonograph* or Ultrasound* or Ultrason* or screening* or scan*):ti,ab,kw (Word variations have been searched) | 180968 |
| #5 | #1 or #2 or #3 or #4 | 186683 |
| #6 | MeSH descriptor: [Cervical Length Measurement] explode all trees | 119 |
| #7 | ((Cervi* NEAR/3 (length* or measurement* or short* or assessment*))):ti,ab,kw (Word variations have been searched) | 1398 |
| #8 | #6 or #7 | 1398 |
| #9 | MeSH descriptor: [Premature Birth] this term only | 2548 |
| #10 | MeSH descriptor: [Infant, Premature] explode all trees | 5924 |
| #11 | (Prematur* or Pre-matur* or Preterm* or Pre-term*):ti,ab,kw (Word variations have been searched) | 36114 |
| #12 | #9 or #10 or #11 | 36114 |
| #13 | #5 AND #8 AND #12 | 419 |
| #14 | (clinicaltrials OR trialsearch):so | 535215 |
| #15 | #13 NOT #14 | 295 |
| #16 | (conference proceeding):pt | 248848 |
| #17 | #15 NOT #16 | **242** |

**MeSH descriptor: [] explode all trees** = term from the MeSH controlled vocabulary, including terms found below this term in the hierarchy
**NEAR/3** = Next to each other, in any order, up to 3 words in between

**:ti,ab,kw** = title, abstract and author keywords

**:pt** = publication type

***** = truncation of word for alternate endings

The websites listed below were visited 14 Oct 2024.

Nothing relevant to the question at issue was found/

| **Source** | **Search terms / Browsing** | **No. of results** | **No. of relevant results** |
| --- | --- | --- | --- |
| **SBU**  [www.sbu.se](http://www.sbu.se/) | Cervix  Preterm birth  Preterm  Premature  Pre-term  Ultrasound | 4  6  14  9  71  42 | 1 relevant:  Mapping of methods for diagnosis and treatment of threatening spontaneous preterm birth – Identification of evidence and scientific knowledge gaps based on systematic reviews Stockholm: Swedish Agency for Health Technology Assessment and Assessment of Social Services (SBU); 2021. SBU Kartlägger 320_2. [accessed Jan 18 2022]. Available from: <https://www.sbu.se/320_2>.  2 relevant of which one a duplicate:  Ljungström E, Möller AC, Bergman L, Ekelund A-C, Hongslo Vala C, Jacobsson B, Kuusela P, Liljegren A, Petzold M, Sjögren P, Svensson M, Wennerholm U-B, Strandell A Titel: Progesterone, cerclage, pessary, or acetylsalicylic acid for prevention of preterm birth in singleton and multifetal pregnancies. Göteborg: Västra Götalandsregionen, Sahlgrenska Universitetssjukhuset, HTA-centrum: 2022. Regional activity-based HTA 2022:127 – 130  2 relevant = duplicates  0 relevant  2 relevant = 2 duplicates  1 relevant = duplicate |
| **Folkehelseinstituttet (Norge)**  <https://www.fhi.no/ku/metodevurdering/> | Visited category Evaluation of methods – Reports | 0 | 0 relevant |
| **Behandlingsrådet (Danmark)**  <https://behandlingsraadet.dk/> | Visited | 0 | 0 relevant |
| **Nationale Kliniske Anbefalinger og Retningslinjer (Danmark)**  <https://www.sst.dk/da/Fagperson/Retningslinjer-og-procedurer/NKA-og-NKR/NKR-og-NKA-efter-omraade> | Visited Other subjects | 0 | 0 relevant |
| **CAMTÖ**  <https://www.regionorebrolan.se/sv/forskning/kontakt-och-organisation/hta-enheten-camto/> | Visited 2020:39 up until 2024:72 | 0 | 0 relevant |

| **HTA Region Stockholm**  <https://www.chis.regionstockholm.se/hta/rapporter/> | Visited 2020:53-2024:03 | 0 | 0 relevant |
| --- | --- | --- | --- |
| **Regional samverkansgrupp HTA**  <https://sydostrasjukvardsregionen.se/samverkansgrupper/hta/genomforda-bedomningar/> | Visited 2020-2021 (no reports after this) | 0 | 0 relevant |
| **HTA Syd**  <https://vardgivare.skane.se/kompetens-utveckling/sakkunniggrupper/hta-skane/#110365> | Visited 2020-2024:1 | 0 | 0 relevant |
| **Medicinska rådet, Region Dalarna**  <https://www.regiondalarna.se/plus/vard/utveckling-och-utbildning/kunskapsstyrning/vetenskapliga-radet/#:~:text=Vetenskapliga%20r%C3%A5det%20inr%C3%A4ttades%202024.,beslut%20i%20%C3%B6vergripande%20medicinska%20fr%C3%A5gor>. | Visited. Published reports missing | - | - |

**Reference lists**

A comprehensive review of reference lists brought four new records.
