# Supplementary material for: Can universal cervical length screening with vaginal progesterone for a short cervix reduce preterm birth? A systematic review and meta‐analyses
Source: Acta Obstet Gynecol Scand. 2026 May 20;105(8):1420–34. doi: 10.1111/aogs.70253 (PMC13356482; doi:10.1111/aogs.70253)
Supplement: Supplementary file 4 — Table S3. PICO. [file AOGS-105-1420-s001.docx]

**Table S3.** PICO

**Question(s) at issue:** Is universal cervical length screening with transvaginal ultrasound in women with singleton pregnancies followed by treatment with vaginal or oral progesterone, with or without additional therapy, when short cervix length is demonstrated, effective in preventing any or spontaneous PTB and does it affect perinatal outcomes?

**PICO:** (*P=Patient I=Intervention C=Comparison O=Outcome*)

| **P** | Women with singleton pregnancies in the first or second trimester. |
| --- | --- |
| **I** | Screening with transvaginal ultrasound and measurement of cervical length followed by treatment with vaginal or oral progesterone, with or without additional therapy, if short cervical length* is demonstrated |
| **C** | No screening with transvaginal ultrasound |
| **O** | Critical for decision making:   - Any preterm birth, any cut off below 37+0 weeks, defined by the authors - Spontaneous preterm birth, any cut off below 37+0 weeks, defined by the authors - Perinatal mortality (intrauterine fetal death and neonatal mortality <7 or <28 days) - Neonatal mortality <7, <28 days - Serious neonatal morbidity (such as bronchopulmonary dysplasia, severe intraventricular haemorrhage, necrotizing enterocolitis, confirmed sepsis, retinopathy of prematurity), individually or as a composite outcome with or without peri/neonatal mortality. |

*As defined by the authors

**Eligibility criteria**

**Study design:**

- Systematic reviews
- Randomised controlled trials
- Non-randomised controlled studies with at least 1,000 screened women

**Language:**

- English, Swedish, Norwegian, Danish

**Publication date:**

- 1980-
- Time limit SR: 2020-

**Planned subgroup analyses**

- Different cut-offs for cervical length
- Women with previous PTB
- Women without previous PTB
- First trimester vs second trimester screening
- Exclusion of studies where the intervention includes more than only progesterone
