# Supplementary material for: Can universal cervical length screening with vaginal progesterone for a short cervix reduce preterm birth? A systematic review and meta‐analyses
Source: Acta Obstet Gynecol Scand. 2026 May 20;105(8):1420–34. doi: 10.1111/aogs.70253 (PMC13356482; doi:10.1111/aogs.70253)
Supplement: Supplementary file 5 — Table S4. Reference lists of included and excluded reports. [file AOGS-105-1420-s007.docx]

**Table S4.** Reference lists of included and excluded reports

**Included reports:**

Figarella A, Chau C, Loundou A, d'Ercole C, Bretelle F. The introduction of a universal transvaginal cervical length screening program is associated with a reduced preterm birth rate. Am J Obstet Gynecol. 2023;228(2):219.e1-.e14. doi: <https://doi.org/10.1016/j.ajog.2022.07.046>.

Melchor Corcostegui I, Unibaso Rodriguez E, Ruiz Blanco N, Nikolova T, Nikolova N, Burgos San Cristobal J, et al. Is mid-trimester cervical length screening effective for reduction of threatened preterm labor? Taiwan J Obstet Gynecol. 2023;62(3):412-6. doi: <https://doi.org/10.1016/j.tjog.2022.09.014>.

Mishra S, Bagga R, Kalra J, Jain V, Dutta S. Routine second trimester cervical length screening in low risk women identified women at risk of a 'very' preterm birth but did not reduce the preterm birth rate: a randomised study from India. J Obstet Gynaecol. 2018;38(6):789-95. doi: <https://doi.org/10.1080/01443615.2017.1419461>.

Saccone G, Maruotti G, Morlando M, Visentin S, De AC, Sarno L, et al. Randomized trial of screening for preterm birth in low-risk women - the preterm birth screening study. Am J Obstet Gynecol MFM. 2024;6(5s):101267.

Son M, Grobman WA, Ayala NK, Miller ES. A universal mid-trimester transvaginal cervical length screening program and its associated reduced preterm birth rate. Am J Obstet Gynecol. 2016;214(3):365.e1-.e5. doi: <https://doi.org/10.1016/j.ajog.2015.12.020>.

Souka AP, Maritsa VA, Eleftheriades M. Screening vs. no screening for preterm delivery in low-risk singleton pregnancies: comparison by propensity score analysis. Arch Gynecol Obstet. 2024;309(1):133-8. doi: <https://doi.org/10.1007/s00404-022-06882-w>.

**Systematic review, no appraisal done, only commented up on:**

Hessami K, D'Alberti E, Mascio DD, Berghella V. Universal cervical length screening and risk of spontaneous preterm birth: a systematic review and meta-analysis. American Journal of Obstetrics & Gynecology MFM. 2024;6(5S):101343. doi: <https://doi.org/10.1016/j.ajogmf.2024.101343>

**Excluded reports:**

Boelig RC, Kripalu V, Chen SL, Cruz Y, Roman A, Berghella V. Utility of follow-up cervical length screening in low-risk women with a cervical length of 26 to 29 mm. Am J Obstet Gynecol. 2021;225(2):179.e1-.e6. doi: <https://doi.org/10.1016/j.ajog.2021.02.027>.

Brown K, Lam CK, Binks M. Short cervix and preterm birth in the top end. Aust N Z J Obstet Gynaecol. 2023;63(4):521-6. doi: <https://doi.org/10.1111/ajo.13676>.

Cahill AG, Odibo AO, Caughey AB, Stamilio DM, Hassan SS, Macones GA, et al. Universal cervical length screening and treatment with vaginal progesterone to prevent preterm birth: a decision and economic analysis. Am J Obstet Gynecol. 2010;202(6):548.e1-.e8. doi: <https://doi.org/10.1016/j.ajog.2009.12.005>.

Conde-Agudelo A, Romero R, da Fonseca E, Hassan SS, Nicolaides KH. Vaginal progesterone decreases the risk of preterm birth and adverse perinatal outcomes in singleton gestations with a midtrimester sonographic short cervix (<=25 mm) and without a history of spontaneous preterm birth. Am J Obstet Gynecol. 2025;05:05. doi: <https://dx.doi.org/10.1016/j.ajog.2025.07.003>.

Crosby D, Miletin J, Semberova J, Daly S. Is routine transvaginal cervical length measurement cost-effective in a population where the risk of spontaneous preterm birth is low? Acta Obstet Gynecol Scand. 2016;95(12):1391-5.

Einerson BD, Grobman WA, Miller ES. Cost-effectiveness of risk-based screening for cervical length to prevent preterm birth. Am J Obstet Gynecol. 2016;215(1):100.e1-.e7. doi: <https://doi.org/10.1016/j.ajog.2016.01.192>.

Erasmus I, Nicolaou E, van Gelderen CJ, Nicolaides KH. Cervical length at 23 weeks' gestation--relation to demographic characteristics and previous obstetric history in South African women. S Afr Med J. 2005;95(9):691-5. doi:

Facco FL, Simhan HN. Short ultrasonographic cervical length in women with low-risk obstetric history. Obstet Gynecol. 2013;122(4):858-62. doi: <https://doi.org/10.1097/aog.0b013e3182a2dccd>.

Granese R, Mantegna S, Mondello S, Amadore D, Imbesi G, Calagna G, et al. Preterm birth: incidence, risk factors and second trimester cervical length in a single center population. A two-year retrospective study. Eur Rev Med Pharmacol Sci. 2017;21(19):4270-7. doi:

Gudicha DW, Romero R, Kabiri D, Hernandez-Andrade E, Pacora P, Erez O, et al. Personalized assessment of cervical length improves prediction of spontaneous preterm birth: a standard and a percentile calculator. Am J Obstet Gynecol. 2021;224(3):288.e1-.e17. doi: <https://doi.org/10.1016/j.ajog.2020.09.002>.

Heath VC, Southall TR, Souka AP, Elisseou A, Nicolaides KH. Cervical length at 23 weeks of gestation: prediction of spontaneous preterm delivery. Ultrasound Obstet Gynecol. 1998;12(5):312-7. doi: <https://doi.org/10.1046/j.1469-0705.1998.12050312.x>.

Hebbar S, Koirala S. Role of mid-trimester transvaginal cervical ultrasound in prediction of preterm delivery. JNMA J Nepal Med Assoc. 2006a;45(164):357-61. doi:

Hebbar S, Samjhana K. Role of mid-trimester transvaginal cervical ultrasound in prediction of preterm delivery. Med J Malaysia. 2006b;61(3):307-11. doi:

Hutcheon JA, Amanda Skoll M, Eastabrook GD, Lim KI. The case for universal cervical length screening to prevent preterm birth: is it strong enough to change practice in Canada? J Obstet Gynaecol Can. 2012;34(12):1184-7. doi: <https://doi.org/10.1016/s1701-2163(16)35467-6>.

Iams JD, Goldenberg RL, Meis PJ, Mercer BM, Moawad A, Das A, et al. The length of the cervix and the risk of spontaneous premature delivery. National Institute of Child Health and Human Development Maternal Fetal Medicine Unit Network. N Engl J Med. 1996;334(9):567-72. doi: <https://doi.org/10.1056/nejm199602293340904>.

Jain S, Kilgore M, Edwards RK, Owen J. Revisiting the cost-effectiveness of universal cervical length screening: importance of progesterone efficacy. Am J Obstet Gynecol. 2016;215(1):101.e1-.e7. doi: <https://doi.org/10.1016/j.ajog.2016.01.165>.

Kuusela P, Jacobsson B, Hagberg H, Fadl H, Lindgren P, Wesstrom J, et al. Second-trimester transvaginal ultrasound measurement of cervical length for prediction of preterm birth: a blinded prospective multicentre diagnostic accuracy study. BJOG. 2021;128(2):195-206. doi: <https://doi.org/10.1111/1471-0528.16519>.

Kuusela P, Jacobsson B, Soderlund M, Bejlum C, Almstrom E, Ladfors L, et al. Transvaginal sonographic evaluation of cervical length in the second trimester of asymptomatic singleton pregnancies, and the risk of preterm delivery. Acta Obstet Gynecol Scand. 2015;94(6):598-607. doi: <https://doi.org/10.1111/aogs.12622>.

Kuusela P, Wennerholm UB, Fadl H, Wesstrom J, Lindgren P, Hagberg H, et al. Second trimester cervical length measurements with transvaginal ultrasound: A prospective observational agreement and reliability study. Acta Obstet Gynecol Scand. 2020;99(11):1476-85. doi: <https://doi.org/10.1111/aogs.13895>.

Leshno M, Meiri H, Maymon R. Cost-effectiveness of universal routine sonographic cervical-length measurement at 19 to 25 weeks' gestation. Am J Obstet Gynecol MFM. 2024;6(5S):101313. doi: <https://doi.org/10.1016/j.ajogmf.2024.101313>.

Liu CZ, Ho N, Nguyen AD, Lehner C, Sekar R, Amoako AA. The risk of preterm delivery and pregnancy outcomes in women with asymptomatic short cervix: a retrospective cohort study. J Matern Fetal Neonatal Med. 2021;34(11):1747-53. doi: <https://doi.org/10.1080/14767058.2019.1647163>.

Maerdan M, Shi C, Zhang X, Fan L. The prevalence of short cervix between 20 and 24 weeks of gestation and vaginal progesterone for prolonging of gestation. J Matern Fetal Neonatal Med. 2017;30(14):1646-9. doi: <https://doi.org/10.1080/14767058.2016.1220528>.

Marotta R. Vaginal progesterone reduces preterm birth rate. Pharm Times. 2017(pagination). doi:

Maymon R, Pekar-Zlotin M, Meiri H, Haklai Z, Gordon ES, Shlichkov G, et al. Change in prevalence of preterm birth in Israel following publication of national guidelines recommending routine sonographic cervical-length measurement at 19-25 weeks' gestation. Ultrasound Obstet Gynecol. 2023;61(5):610-6. doi: <https://doi.org/10.1002/uog.26093>.

McCurdy RJ, Baxter JK. Universal cervical length screening with a cervicometer to prevent preterm birth <34 weeks: a decision and economic analysis. J Matern Fetal Neonatal Med. 2020;33(21):3670-9. doi: <https://doi.org/10.1080/14767058.2019.1583202>.

Miller ES, Tita AT, Grobman WA. Second-Trimester Cervical Length Screening Among Asymptomatic Women: An Evaluation of Risk-Based Strategies. Obstet Gynecol. 2015;126(1):61-6. doi: <https://doi.org/10.1097/aog.0000000000000864>.

Navathe R, Saccone G, Villani M, Knapp J, Cruz Y, Boelig R, et al. Decrease in the incidence of threatened preterm labor after implementation of transvaginal ultrasound cervical length universal screening. J Matern Fetal Neonatal Med. 2019;32(11):1853-8. doi: <https://doi.org/10.1080/14767058.2017.1421166>.

Newnham JP, White SW, Meharry S, Lee HS, Pedretti MK, Arrese CA, et al. Reducing preterm birth by a statewide multifaceted program: an implementation study. Am J Obstet Gynecol. 2017;216(5):434-42. doi: <https://doi.org/10.1016/j.ajog.2016.11.1037>.

Orzechowski KM, Boelig R, Nicholas SS, Baxter J, Berghella V. Is universal cervical length screening indicated in women with prior term birth? Am J Obstet Gynecol. 2015;212(2):234.e1-.e5. doi: <https://doi.org/10.1016/j.ajog.2014.08.029>.

Orzechowski KM, Boelig RC, Baxter JK, Berghella V. A universal transvaginal cervical length screening program for preterm birth prevention. Obstet Gynecol. 2014a;124(3):520-5. doi: <https://doi.org/10.1097/aog.0000000000000428>.

Orzechowski KM, Nicholas SS, Baxter JK, Weiner S, Berghella V. Implementation of a universal cervical length screening program for the prevention of preterm birth. Am J Perinatol. 2014b;31(12):1057-62. doi: <https://doi.org/10.1055/s-0034-1371710>.

Rawashdeh H, Ramachandran A, Yang JM, Blain G, Hyett J. Changing Indications for Cervical Cerclage Following the Introduction of Routine Ultrasound Surveillance of Cervical Length for Prediction and Prevention of Preterm Birth. Int J Women Health. 2024;16:1755-64. doi: <https://dx.doi.org/10.2147/IJWH.S477974>.

Romero R, Conde-Agudelo A, da Fonseca E, O'Brien JM, Creasy GW, Hassan SS, et al. Vaginal progesterone reduces the risk of preterm birth and adverse perinatal outcomes in singleton gestations with a midtrimester sonographic short cervix (<=25 mm): an updated individual patient data meta-analysis. Am J Obstet Gynecol. 2025;233(1):e1-e5. doi: <https://dx.doi.org/10.1016/j.ajog.2025.03.013>.

Romero R. Spontaneous preterm labor can be predicted and prevented. Ultrasound Obstet Gynecol. 2021;57(1):19-21. doi: <https://doi.org/10.1002/uog.23565>.

Rosenbloom JI, Raghuraman N, Temming LA, Stout MJ, Tuuli MG, Dicke JM, et al. Predictive Value of Midtrimester Universal Cervical Length Screening Based on Parity. J Ultrasound Med. 2020;39(1):147-54. doi: <https://doi.org/10.1002/jum.15091>.

Sandager KP, Vogel I, Thorsen P, Uldbjerg N. [Cervical length as a predictor of preterm delivery]. Ugeskr Laeger. 2003;165(46):4415-8. doi:

Schlembach D. Cervical length and premature birth: Importance of ultrasound measurement. Gynakologische Praxis. 2017;41(4):575-8. doi:

Seravalli V, Abati I, Strambi N, Tofani L, Tucci C, Tartarotti E, et al. Universal cervical length screening for preterm birth is not useful after 24 weeks of gestation. Acta Obstet Gynecol Scand. 2023;102(11):1541-8. doi: <https://doi.org/10.1111/aogs.14683>.

Shainker SA, Modest AM, Hacker MR, Ralston SJ. The Effect of a Universal Cervical Length Screening Program on Antepartum Management and Birth Outcomes. AJP Rep. 2016;6(2):e206-e11. doi: <https://doi.org/10.1055/s-0036-1584240>.

Silva T, Borovac-Pinheiro A, Pacagnella R. Estimates of avoided costs attributed to a short cervix screening program to prevent preterm birth from the perspective of the Unified Health System (SUS). Rev Saude Publica. 2023;57:87.

Slager J, Lynne S. Short Cervix and the Risk of Preterm Birth. J Midwifery Womens Health. 2012;57(SUPPL.1):19-20. doi: <https://doi.org/10.1111/j.1542-2011.2012.00211.x>.

Son M, Miller ES. Predicting preterm birth: Cervical length and fetal fibronectin. Semin Perinatol. 2017;41(8):445-51. doi: <https://doi.org/10.1053/j.semperi.2017.08.002>.

Soto-Torres EE, Hernandez-Andrade E, Huntley ES, Blackwell SC. Maternal and obstetrical factors associated with short cervical length at midtrimester in women with no history of preterm delivery. J Matern Fetal Neonatal Med. 2023;36(2):2228448. doi: <https://doi.org/10.1080/14767058.2023.2228448>.

Souka AP, Papastefanou I, Pilalis A, Kassanos D, Papadopoulos G. Implementation of universal screening for preterm delivery by mid-trimester cervical-length measurement. Ultrasound Obstet Gynecol. 2019;53(3):396-401. doi: <https://doi.org/10.1002/uog.19050>.

Stratulat V, Melamed N, Barrett J, Ladhani NNN, Anabusi S, Quaglietta P, et al. Cervical assessment certification and its impact on performance quality in the context of universal cervical screening. Int J Gynaecol Obstet. 2024;164(3):951-8. doi: <https://doi.org/10.1002/ijgo.15078>.

Taipale P, Hiilesmaa V. Sonographic measurement of uterine cervix at 18-22 weeks' gestation and the risk of preterm delivery. Obstet Gynecol. 1998;92(6):902-7. doi: <https://doi.org/10.1016/s0029-7844(98)00346-9>.

Temming LA, Durst JK, Tuuli MG, Stout MJ, Dicke JM, Macones GA, et al. Universal cervical length screening: implementation and outcomes. Am J Obstet Gynecol. 2016a;214(4):523.e1-.e8. doi: <https://doi.org/10.1016/j.ajog.2016.02.002>.

Temming LA, Macones GA. What is prenatal screening and why to do it? Semin Perinatol. 2016b;40(1):3-11. doi: 10.1053/j.semperi.2015.11.002.

van Gils L, Bosmans JE, de Haan-Jebbink JM, Pajkrt E, Oudijk MA. Budget impact analysis of cervical length measurement during the routine second trimester anomaly scan for the prevention of spontaneous preterm birth in the Netherlands. Eur J Obstet Gynecol Reprod Biol. 2025;312:114550. doi: <https://dx.doi.org/10.1016/j.ejogrb.2025.114550>.

Werner EF, Hamel MS, Orzechowski K, Berghella V, Thung SF. Cost-effectiveness of transvaginal ultrasound cervical length screening in singletons without a prior preterm birth: an update. Am J Obstet Gynecol. 2015;213(4):554.e1-.e6. doi: <https://doi.org/10.1016/j.ajog.2015.06.020>.

Werner EF, Han CS, Pettker CM, Buhimschi CS, Copel JA, Funai EF, et al. Universal cervical-length screening to prevent preterm birth: a cost-effectiveness analysis. Ultrasound Obstet Gynecol. 2011;38(1):32-7. doi: <https://doi.org/10.1002/uog.8911>.

Wikstrom T, Hagberg H, Jacobsson B, Kuusela P, Wesstrom J, Lindgren P, et al. Effect of second-trimester sonographic cervical length on the risk of spontaneous preterm delivery in different risk groups: A prospective observational multicenter study. Acta Obstet Gynecol Scand. 2021;100(9):1644-55. doi: <https://doi.org/10.1111/aogs.14203>.

Wikstrom T, Kuusela P, Jacobsson B, Hagberg H, Lindgren P, Svensson M, et al. Cost-effectiveness of cervical length screening and progesterone treatment to prevent spontaneous preterm delivery in Sweden. Ultrasound Obstet Gynecol. 2022;59(6):778-92. doi: <https://doi.org/10.1002/uog.24884>.

Williams M, Iams JD. Cervical length measurement and cervical cerclage to prevent preterm birth. Clin Obstet Gynecol. 2004;47(4):775-83. doi: <https://doi.org/10.1097/01.grf.0000141895.85221.be>.

Wu T, Li S, Gong X, Li J, Li X, Zhai Y, et al. Longitudinal Cervical Length Measurements and Spontaneous Preterm Birth in Singleton and Twin Pregnancies. JAMA Network Open. 2024;7(4):e244592. doi: <https://doi.org/10.1001/jamanetworkopen.2024.4592>.

Wulff CB, Rode L, Rosthoj S, Hoseth E, Petersen OB, Tabor A. Transvaginal sonographic cervical length in first and second trimesters in a low-risk population: a prospective study. Ultrasound Obstet Gynecol. 2018;51(5):604-13. doi: <https://doi.org/10.1002/uog.17556>.
