# Supplementary material for: Can universal cervical length screening with vaginal progesterone for a short cervix reduce preterm birth? A systematic review and meta‐analyses
Source: Acta Obstet Gynecol Scand. 2026 May 20;105(8):1420–34. doi: 10.1111/aogs.70253 (PMC13356482; doi:10.1111/aogs.70253)
Supplement: Supplementary file 6 — Table S5. Excluded articles with reasons for exclusion. [file AOGS-105-1420-s005.docx]

**Table S5.** Excluded articles and reasons for exclusion

| **Author, year** | **Reason for exclusion** |
| --- | --- |
| Boelig, 2021 | Wrong comparison (comparison with no screening is missing) |
| Brown, 2023 | Wrong comparison (comparison with no screening is missing) |
| Cahill, 2010 | Wrong study design (decision-analysis model) |
| Conde-Agudelo, 2025 | Wrong study design (no screening) |
| Crosby, 2016 | Wrong study design (decision-analysis model) |
| Einerson, 2016 | Wrong study design (decision-analysis model) |
| Erasmus, 2005 | Wrong comparison (comparison with no screening is missing) |
| Facco, 2013 | Wrong comparison (comparison with no screening is missing) |
| Granese, 2017 | Wrong comparison (comparison with no screening is missing) |
| Gudicha, 2021 | Wrong comparison (comparison with no screening is missing) |
| Heath, 1998 | Wrong comparison (comparison with no screening is missing) |
| Hebbar, 2006a | Wrong comparison (comparison with no screening is missing) |
| Hebbar, 2006b | Wrong comparison (comparison with no screening is missing) |
| Hutcheon, 2012 | Wrong publication type (commentary) |
| Iams, 1996 | Wrong comparison (comparison with no screening is missing) |
| Jain, 2016 | Wrong study design (decision-analysis model) |
| Kuusela, 2015 | Wrong intervention |
| Kuusela, 2020 | Wrong focus (estimate inter- and intraobserver agreement and reliability) |
| Kuusela, 2021 | No intervention |
| Leshno, 2024 | Wrong study design (decision-analysis model) |
| Liu, 2021 | Wrong population (third trimester included) |
| Maerdan, 2017 | Wrong comparison (comparison with no screening is missing) |
| Marotta, 2017 | Wrong population (twins) |
| Maymon, 2023 | Wrong intervention (primary screening with transabdominal ultrasound) |
| McCurdy, 2020 | Wrong study design (decision-analysis model) |
| Miller, 2015 | Wrong comparison (comparison with no screening is missing) |
| Navathe, 2019 | Wrong outcome (preterm labour not birth) |
| Newnham, 2017 | Wrong intervention (primary screening with transabdominal ultrasound) |
| Orzechowski, 2014a | Wrong control group (subjects declining screening) |
| Orzechowski, 2014b | Wrong focus (evaluation of implementation and acceptability of universal screening) |
| Orzechowski, 2015 | Wrong comparison (comparison with no screening is missing) |
| Rawashdeh, 2024 | Wrong intervention (cerclage) |
| Romero, 2025 | Wrong study design (no screening) |
| Romero, 2021 | Wrong publication type (opinion) |
| Rosenbloom, 2020 | Wrong comparison (comparison with no screening is missing) |
| Sandager, 2003 | Wrong study design (review, not systematic) |
| Schlembach, 2017 | Wrong language (German) |
| Serrvalli, 2023 | Wrong comparison (comparison with no screening is missing) |
| Shainker, 2016 | Wrong intervention (cerclage without progesterone) |
| Silva, 2023 | Wrong study design (decision-analysis model) |
| Slager, 2012 | Wrong publication type (summary) |
| Son, 2017 | Wrong study design (review, not systematic) |
| Soto-Torres, 2023 | Wrong comparison (comparison with no screening is missing) |
| Souka, 2019 | Duplicate publication (Souka 2024) |
| Stratulat, 2024 | Wrong control (screening with transabdominal ultrasound) |
| Taipale, 1998 | Wrong comparison (comparison with no screening is missing) |
| Temming, 2016a | Wrong control group (subjects declining screening) |
| Temming, 2016b | Wrong study design (review, not systematic) |
| Van Gils, 2025 | Wrong study design (decision-analysis model) |
| Werner, 2011 | Wrong study design (decision-analysis model) |
| Werner, 2015 | Wrong study design (decision-analysis model) |
| Wikstrom, 2021 | Wrong comparison (comparison with no screening is missing) |
| Wikström, 2022 | Wrong study design (decision-analysis model) |
| Williams, 2004 | Wrong intervention (cerclage without progesterone) |
| Wu, 2024 | Wrong comparison (comparison with no screening is missing) |
| Wulff, 2018 | Wrong control group (subjects declining screening) |
