# Supplementary material for: Can universal cervical length screening with vaginal progesterone for a short cervix reduce preterm birth? A systematic review and meta‐analyses
Source: Acta Obstet Gynecol Scand. 2026 May 20;105(8):1420–34. doi: 10.1111/aogs.70253 (PMC13356482; doi:10.1111/aogs.70253)
Supplement: Supplementary file 7 — Table S6. Specification of aspects important for quality assessment. [file AOGS-105-1420-s006.docx]

**Table S6.** Specification of aspects important for quality assessment

*Cont.*

| **Author**  **Year**  **Country** | **Study design** | **Problems contributing to downgrading the study in the assessment^1^** | | | | | | |
| --- | --- | --- | --- | --- | --- | --- | --- | --- |
|  |  | **Directness** | | **Study limitations** | | **Precision** | | |
| Mishra et al.  2018  India | RCT | ?/- | Ethnicity  High level of high-risk patients  Unclear clinical setting/context  Unclear selection process | ? | Unclear if the allocation is concealed  Unclear where the finance of the study comes from.  Not blinded assessments  No certification for ultrasound staff  Important variables are missing (e.g. smoking, BMI)  Unclear if the patient groups have been treated equally  Protocol maybe registered after publication and not easily available  Side effects not reported | - | Few events  Power calculation not reasonable Power does not match the result at all | |
| Saccone et al.  2024  Italy | RCT | + |  | ? | Not blinded assessments  Compliance and reasons for lost to follow-up not reported | - | Few events  Power calculation too optimistic (50% reduction of PTB)  Power does not match the results | |
| Figarella et al.  2023  France | Cohort  before-after | ? | Unclear ethnicity  Difficult to evaluate the effect with so many screened in the comparison group (28.9%) - however relevant in a transition phase in Sweden  Unclear number of women treated with progesterone | ?/- | Not blinded assessments  Baseline differences  No statistical handling of group imbalances  Important variables are missing  Unclear compliance  Not adjusted for any variables  Side effects not reported | + |  | |
| Melchor et al.  2023  Spain | Cohort  before-after | - | Unclear study population (high-risk patients not reported, no info on base population, only demographics for the small preterm labour group) | - | Not blinded assessments  Confounders and finance not reported | ? | Power calculations not reported Primary outcome not our outcome | |
| Son et al.  2016  US | Cohort  before-after | + |  | ? | Not blinded assessments  Baseline differences  Finance not reported  Low treatment rate with progesterone | + |  | |
| **Author**  **Year**  **Country** | **Study design** | **Problems contributing to downgrading the study in the assessment^1^** | | | | | | |
|  |  | **Directness** | | **Study limitations** | | **Precision** | | |
| Souka et al.  2024  Greece | Cohort propensity  score matched | ? | Private hospitals  Unclear study population (high-risk patients not reported, missing important info)  Unclear intervention (obstetrician decides treatment, limited cut-off to  15 mm) | - | Not blinded assessments  Risk of selection bias when choosing the control group  Large dropout in the screening group  Compliance not reported  Unclear if the correct statistical methods were used (Hazard ratio) | - | | Power calculations not reported  Few events |

1 Aspects regarding directness and study limitations identified during the assessment process contributing to the study being categorised as having no/minor (+), some (?) or major (-) problems. These assessments applied to all outcomes if not explicitly stated otherwise.

BMI: Body mass index, PTB: preterm birth, RCT: randomised controlled trial, US: United States
