# Supplementary material for: Can universal cervical length screening with vaginal progesterone for a short cervix reduce preterm birth? A systematic review and meta‐analyses
Source: Acta Obstet Gynecol Scand. 2026 May 20;105(8):1420–34. doi: 10.1111/aogs.70253 (PMC13356482; doi:10.1111/aogs.70253)
Supplement: Supplementary file 8 — Table S7. Outcome tables A‐D. [file AOGS-105-1420-s002.docx]

**S7 Table.** Outcome tables A-D

**A.** Any preterm birth

*Cont.*

| **Author**  **year**  **country** | **Study design** | **Number of patients** | **Withdrawals** **-** **dropouts** | **Results** | | | **Comments** | **Directness *** | **Study limitations *** | **Precision *** |
| --- | --- | --- | --- | --- | --- | --- | --- | --- | --- | --- |
|  |  |  |  | **Intervention (%)** | **Control**  **n (%)** | **Difference** |  |  |  |  |
| Saccone  2024  Itay | RCT | n=1.334  I=675  C=659 | I=36  C=41  Eligible: 1487  Excluded before enrolment: 153 (including declined 69) | < 37w: 48/639 (7.5)  < 34w: 14/639 (2.2)  < 32w: 9/639 (1.4)  < 30w: 6/639 (0.9)  < 28w: 3/639 (0.5)  < 24w: 0/639 | < 37w: 54/618 (8.7)  < 34w: 14/618 (2.3)  < 32w: 10/618 (1.6)  < 30w: 7/618 (1.1)  < 28w: 5/618 (0.8)  < 24w: 2/618 (0.3) | **RR (CI 95%), p value**  0.86 (0.59-1.25), 0.43  0.97 (0.46-2.01), 0.93  0.87 (0.36-2.13), 0.76  0.83 (0.28-2.45), 0.72  0.58 (0.14-2.42), 0.45  0.19 (0.01-4.02), 0.29 | **-** | + | ? | ? |
| Figarella  2023  France | Cohort before-after | n=336,603  Period A  C=171,079  (not screened)  Period B  I=165,524  (screened) | - | Period B  < 37w:  9275/165,524 (5.60) | Period A  < 37w:  9903/171,079 (5.79) | **aOR (95% CI), p value**  0.92 (0.89-0.95), <0.0001 | Study with 2 parts, prospective cohort (ECHOCOL) not included in analysis.  Variables used in adjusted analysis not shown, only described as “variables that had clinical relevance” | ? | ?/- | + |
| Son  2016  US | Cohort before-after | n=64,214  I=17,616  (screened)  C=46,598  (not screened) | I: 7 terminated their pregnancies after screening, excluded from analysis.  19 declined screening, included in analysis. | < 37w: 1051/17,609 (6.0)  < 34w: 291/17,609 (1.7)  < 32w: 168/17,609 (1.0) | < 37w: 3141/46,598 (6.7)  < 34w: 907/46,598 (1.9)  < 32w: 532/46,598 (1.1) | **OR (95% CI) p-value**  **aOR (95% CI)**  0.88 (0.82-0.94)  p<0.001  0.82 (0.76-0.88)  0.85 (0.74-0.97)  p=0.014  0.74 (0.64-0.85)  0.84 (0.70-0.99)  p=0.041  0.74 (0.62-0.90) | Adjusted for race/  ethnicity, BMI,  history of cervical excision, smoking, chronic hypertension and pre-gestational diabetes | + | ? | + |

aOR: adjusted odds ratio, BMI: body mass index, C: control, CI: confidence interval, I: intervention, n: number, OR: odds ratio, RCT: randomized controlled trial, RR: risk ratio, US: United States, w: weeks


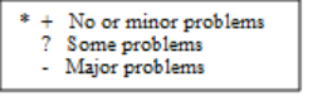


*Cont.*

**B.** Spontaneous preterm birth

*Cont.*

| **Author year**  **country** | **Study design** | **Number** **of patients** **n=** | **Withdrawals-** **dropouts** | **Results** | | | **Comments** | **Directness *** | **Study limitations *** | **Precision *** |
| --- | --- | --- | --- | --- | --- | --- | --- | --- | --- | --- |
|  |  |  |  | **Intervention**  **n (%)** | **Control**  **n (%)** | **Difference** |  |  |  |  |
| Mishra  2018  India | RCT | n=300  I=150  C=150 | I=3 (2 induced PTB, 1 lost to follow up)  C=1 (induced PTB)  Eligible: 1113  Excluded before enrolment: 813 (no decliners) | <37w: 15/147 (10.2)  Subgroups (secondary outcome):  36+6 –32+0w:  13/147 (8.84)  28+0 - <32w:  2/147 (1.36)  <28: 0/147 | <37w: 12/149 (8.1)  Subgroups (secondary outcome:  36+6 –32+0w:  11/149 (7.3)  28+0 - <32w:  1/149 (0.7)  <28: 0/149 | **p value**  0.433      p-value for stratification of preterm birth subgroups 0.370 | 73% excluded before enrolment. | ?/- | ? | - |
| Saccone  2024  Italy | RCT | n=1.334  I=675  C=659 | I=36  C=41  Eligible: 1.487  Excluded before enrolment: 153 (including declined 69) | < 37w: 36/639 (5.6)  < 34w: 9/639 (1.4)  < 32w: 6/639 (0.9)  < 30w: 4/639 (0.6)  < 28w: 2/639 (0.3)  < 24w: 0/639 | < 37w: 39/618 (6.3)  < 34w: 12/618 (1.9)  < 32w: 8/618 (1.3)  < 30w: 5/618 (0.8)  < 28w: 3/618 (0.5)  < 24w: 1/618 (0.2) | **RR (CI 95%), p value**  0.89 (0.58-1.39), 0.61  0.73 (0.31-1.71), 0.46  0.73 (0.25-2.08), 0.55  0.77 (0.21-2.87), 0.70  0.64 (0.11-3.85), 0.63  0.32 (0.01-7.90), 0.49 |  | + | ? | ? |
| Figarella  2023  France | Cohort  before-after | n=336, 603  Period A  C=171, 079  before screening  Period B  I=165, 524  after screening | - | **Period B**  <37w:  2976/165,524 (1.8) | **Period A**  <37w**:**  3262/171,079 (1.9) | **OR 95% CI, p value**  0.94 (0.88-0.99), 0.02 | During period A  49,504/171,079 (28.9%) were screened  During period B  87.546/165.524 (52.9%) were screened | ? | ?/- | + |
| Melchor Corcostegui  2023  Spain | Cohort  before-after | n=1110  I=628  screened  C=482  not screened | I=145  C=72  Eligible: 1110  Excluded before enrolment: 128  (I: 103 C: 25)  Excluded after enrolment: 89  (I: 42 C: 47), abandon or repeated visits | <37w: 77/483 (15.94)  ≤35w: 59/483 (12.21)  ≤34w: 35/483 (7.24)  ≤32w: 12/483 (2.48)  ≤28w: 4/483 (0.82) | <37w: 105/410 (25.60)  ≤35w: 80/410 (19.51)  ≤34w: 44/410 (10.73)  ≤32w: 13/410 (3.17)  ≤28w: 3/410 (0.73) | **p value**  0.0004  0.0027  0.0673  0.5334  0.8789 |  | - | - | ? |
| Son  2016  US | Cohort before-after | n=81,816  I=17,609  C=64,207 | I: 7 terminated their pregnancies after screening, not included in analysis.  19 declined screening, included in analysis | <37w: 701/17,609 (4.0)  <34w: 176/17,609 (1.0)  <32w: 94/17,609 (0.5) | <37w: 2258/46,598 (4.8)  <34w: 594/46,598 (1.3)  <32w: 328/46,598 (0.7) | **OR (95% CI)**  **aOR (95% CI)**  0.81 (0.75-0.89)  p<0.001  0.79 (0.72-0.86)  0.78 (0.66-0.93)  p=0.004  0.72 (0.60-0.86)  0.76 (0.60-0.95)  p=0.017  0.70 (0.55-0.89) | Adjusted for race/ethnicity, BMI, history of cervical excision, smoking, chronic hypertension and pregestational diabetes | + | ? | + |
| Souka  2024  Greece | Cohort  Propen-sity score match-ed | n= 6206  I=3103  screened  C=3103  not screened | Eligible: 10,133  (I: 6913, C: 3220)  Excluded: 3927 after propensity score matching | 24-32w: 10/3103 (0.3)  20-32w: 10/3103 (0.3) | 24-32w: 25/3103 (0.8)  20-32w: 27/3103 (0.9) | **HR (95% CI), p value**  0.39 (0.19-0.82), 0.013    0.36 (0.18-0.75), 0.006 |  | ? | - | ? |

aOR: adjusted odds ratio, C: comparison, CI: confidence interval, HR hazard ratio, I: intervention, n: number, OR: odds ratio, PTB: preterm birth, RCT: randomized controlled study, RR: risk ratio, US: United States, w: weeks

*Cont.*

**C.** Serious neonatal morbidity

*Cont.*

| **Author**  **year**  **country** | **Study design** | **Number** **of patients** **n=** | **Withdrawals** **-** **dropouts** | **Results** | | | **Comments** | **Directness *** | **Study limitations *** | **Precision *** |
| --- | --- | --- | --- | --- | --- | --- | --- | --- | --- | --- |
|  |  |  |  | **Intervention** | **Control** | **Difference** |  |  |  |  |
| Mishra  2018  India,  one tertiary centre | RCT | I: 150  screened  C: 150  not screened | I:3  (one  lost to follow up, two iatrogenic PTBs)  C: 1  (one iatrogenic PTB) | Neonates with complications*  21/147 (14.3%)  Neonates with complications  including NNM*  21/147 (14.3%)  RDS 4*/147 (2.7%)  IVH 1*/147 (0.7%) | Neonates with complications*  16/149 (10.7%)  Neonates with complications  including NNM*  18/149 (12.1%)  RDS 2/149 (1.3%)  IVH 2/149 (1.3%) | p=0.187  p=0.554  p=0.552 | *Neonates with complications include at least one of neonatal jaundice, RDS, IVH or neonatal jaundice without or with NNM. Neonatal complications calculated from table 3.  **One neonate had RDS and IVH | ?/- | ? | - |
| Saccone  2024  Italy,  2 centres | RCT | I: 675  screened  C: 659  not screened | I: 36  C: 41 | Composite perinatal outcome including  NNM*  19/639 (3.0%)  NEC 0/639  IVH 3-4, 1/639 (0.2%)  RDS 12/639 (1.9%)  BPD 1/639 (0.2%)  ROP 0/639  Sepsis 6/639 (0.9%) | Composite perinatal outcome including NNM*  26/618 (4.2%)  NEC  1/618 (0.2%)  IVH 3-4  2/618 (0.3%)  RDS 19/618 (3.1%)  BPD 3/618 (0.5%)  ROP 3/618 (0.5%)  Sepsis 9/618 (1.4%) | RR 0.71 (95% CI 0.40-1.26)  p=0.24  RR 0.32 (95% CI 0.01-7.90)  p=0.49  RR 0.48 (95% CI 0.04-5.32)  p=0.55  RR 0.61 (95% CI 0.30-1.25), p=0.18  RR 0.32 (95% CI 0.03-3.09) p=0.33  RR 0.14 (95% CI 0.01-2.67) p=0.19  RR 0.64 (95% CI 0.23-1.80) p=0.40 | *Composite perinatal outcome includes at least one of NEC, IVH 3-4, RDS, BPD, ROP, proven sepsis, or NNM | + | ? | ?  *Cont.* |

BPD: bronchopulmonary dysplasia, C: control, CI: confidence interval, I: intervention, IVH: intraventricular haemorrhage, IUFD: intrauterine fetal death, NEC: necrotizing enterocolitis, NNM: neonatal mortality, RCT: randomized controlled study, RDS: respiratory distress syndrome, ROP: retinopathy of prematurity; RR: risk ratio


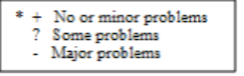


*Cont.*

**D.** Intrauterine foetal death, neonatal mortality

| **Author**  **year**  **country** | **Study design** | **Number** **of patients** **n=** | **Withdrawals** **-** **dropouts** | **Results** | | | **Comments** | **Directness *** | **Study limitations *** | **Precision *** |
| --- | --- | --- | --- | --- | --- | --- | --- | --- | --- | --- |
|  |  |  |  | **Intervention** | **Control** | **Difference** |  |  |  |  |
| Mishra  2018  India,  one tertiary centre | RCT | I: 150  screened  C: 150  not screened | I: 3  (one lost to follow up, two iatrogenic PTBs)  C: 1  (one iatrogenic PTB) | NNM 0/147 | NNM 2/149 | p=0.552 | NNM  not defined | ?/- | ? | - |
| Saccone  2024  Italy,  2 centres | RCT | I: 675  screened  C: 659  not screened | **-** | IUFD 1/639 (0.2%)  NNM 3/639 (0.5%) | IUFD 1/618 (0.2%)  NNM  4/618 (0.6%) | RR 0.97 (95% CI 0.06- 15.43) p=0.98  RR 0.73 (95% CI 0.16-3.23)  p=0.67 | NNM  (<28 days) | + | ? | ? |


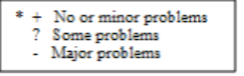
C: control, CI: confidence interval, I: intervention, IUFD: intrauterine fetal death, NNM: neonatal mortality, PTB: preterm birth, RCT: randomised controlled study, RR: risk ratio
